# Efficient material selection for training occluded mmWave radar-based gesture recognisers

**DOI:** 10.1038/s41598-026-56018-2

**Published:** 2026-06-13

**Authors:** Nuwan T. Attygalle, Luis A. Leiva, Matjaž Kljun, Klen Čopič Pucihar

**Affiliations:** 1 Université catholique de Louvain, Louvain, Belgium; 2 University of Luxembourg, Esch-sur-Alzette, Luxembourg; 3 University of Primorska, Koper, Slovenia; 4 Department of Information Science, Stellenbosch University, Stellenbosch, South Africa

**Keywords:** Millimetre-wave radar, Gesture recognition, Material selection, Sampling, Synthetic noise, Engineering, Materials science

## Abstract

Radar-based gesture recognition has emerged as a promising approach for unobtrusive interaction. Unlike camera-based systems, radar sensors can detect gestures through opaque materials, enabling seamless embedding into various everyday objects. However, it remains unclear how to train models efficiently for robust gesture recognition through diverse materials. To investigate this, we collected a dataset of 17,520 gesture recordings performed through 73 everyday materials. By comparing several material-sampling and data-augmentation strategies, we found that a small carefully selected representative subset of training materials was sufficient to match the performance of a classifier trained on the full material dataset. Our results showed that the models trained on 14 quota-sampled materials achieved accuracies of 95.8% and 91.2%, comparable to training on all 73 materials (96.8% and 91.6%) and significantly better than training without material data (66.8% and 65.8%). Among the evaluated sampling approaches, Quota sampling also provided the best overall trade-off between performance and practicality. In contrast, classifiers trained on augmented data performed worse than those trained on actual material-specific data. Taken together, these findings indicate that, for the tested sensor, gesture set, and material collection, carefully selected real-material data offer a practical route to reducing material-specific data collection in radar-based gesture recognition while preserving generalisation. Code, models, and data are available in the public repository, with additional details provided in the supplementary materials: https://gitlab.com/hicuplab/seeing-through.

## Introduction

While traditional interfaces such as keyboards, mice, and touchscreens remain prevalent, alternative modalities are broadening the Human-Computer Interaction (HCI) landscape. Among them, mid-air gesture recognition is gaining popularity due to its intuitive nature, contactless control, and potential for seamless integration into pervasive computing environments. However, gesture sensing systems face challenges with feedback, contextual awareness, and robustness under real-world conditions^1^, especially when the sensing hardware is embedded in everyday objects^2^.

Embedding radar sensors introduces added complexity, as the radar signal must propagate through the object’s material twice (on transmission and on reflection) before reaching the receiving antenna. Signal attenuation depends on the material’s electromagnetic properties, particularly its transmission coefficient ($T_c$). Although prior work has shown that material properties affect signal quality and recognition accuracy^3,4^, these studies often examined only a few materials or overlooked the effect of training with material-specific data.

Deep learning classifiers typically improve with exposure to a larger and more diverse training set, until a saturation point is reached^5–8^. Accordingly, a gesture classifier trained on gesture recordings of a radar signal captured through a wide range of materials (material-specific data) is expected to outperform a classifier trained with limited or no materials. However, re-training a classifier each time the gesture set changes is costly, as it requires re-capturing data through all materials. This highlights the need for more efficient training strategies, which is an open research challenge.

To this end, we conducted a systematic study on radar-based gesture recognition through materials, aiming to optimise the training process. Specifically, we investigate how to train deep learning models that are both accurate and resource-efficient by identifying a minimal yet representative set of training materials. Prior work^3^ has shown that the transmission coefficient is a strong and practical descriptor for predicting performance degradation through materials and is easier to acquire than alternatives such as Signal to Noise Ratio (SNR) (see Section The effect of materials on radar signal quality^3^;). Building on these findings, we hypothesise that $T_c$ can guide material selection.

**Fig. 1:**
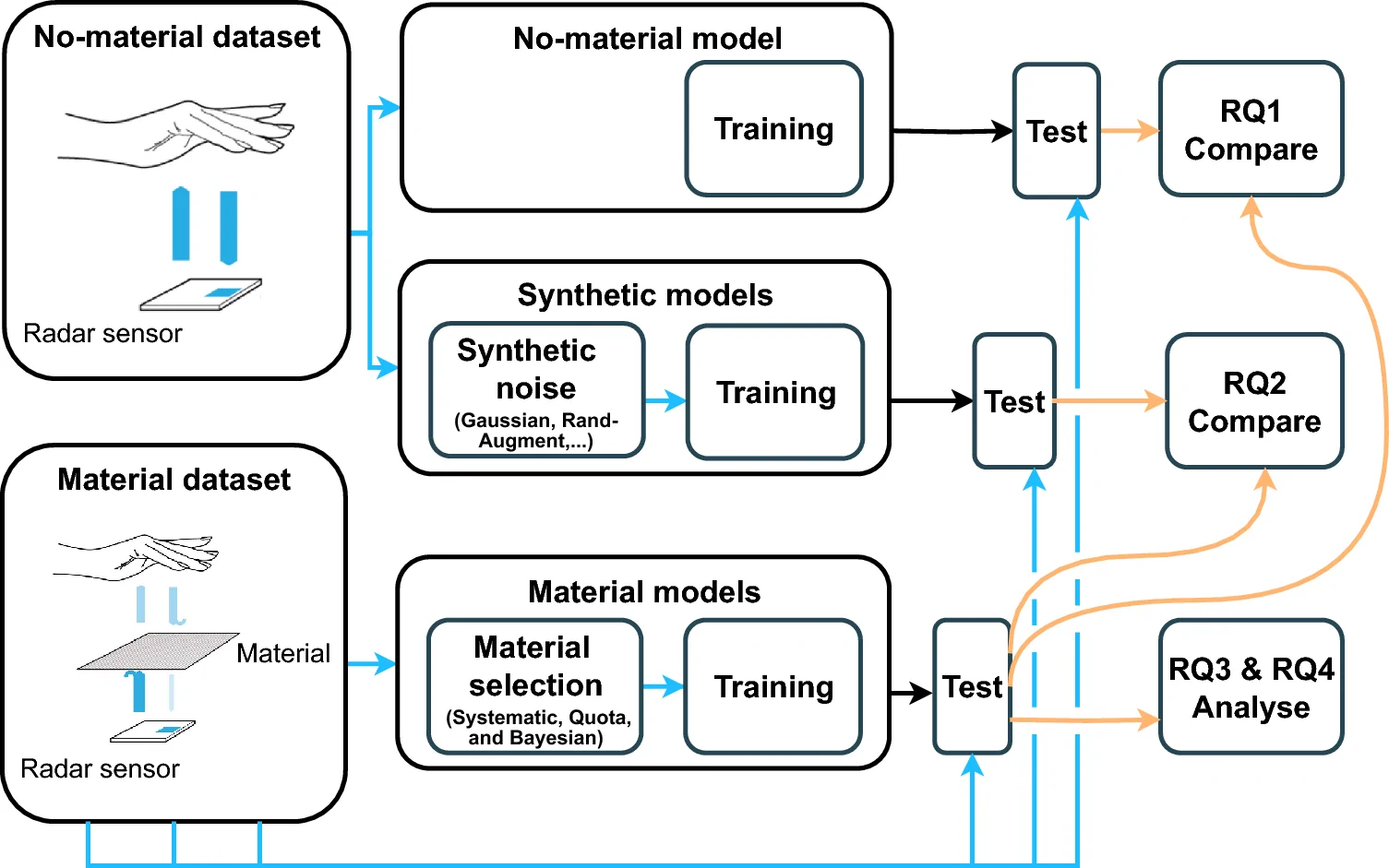
Methodology overview of radar-based gesture recognition through materials.

Using a millimetre-wave (mmWave) radar sensor (Google Soli), we collected a comprehensive dataset of 17,520 gesture recordings through 73 everyday materials. Six gestures were each repeated 40 times per material (i.e., *Material dataset*). We evaluated several training strategies, including synthetic noise injection and three sampling techniques (systematic, quota, and Bayesian) each applied with two objective functions (i.e., *Significance-based* and *Multiple-criteria-based*) for determining the optimal number of training materials.  The experiments follow the methodology presented in Fig. 1 and address the following research questions:

### RQ1:

Does training with the *Material dataset* improve performance?

### RQ2:

Can training with synthetic noise injected into *No-material dataset* achieve comparable performance to training with the *Material dataset*?

### RQ3:

How can training materials be intelligently selected?

### RQ4:

How few training materials are needed to achieve comparable performance to training on all available materials in the *Material dataset*?

Figure 1 summarises our overall methodology, including the no-material, synthetic-noise, and material-specific training pipelines, and how these relate to RQ1–RQ4. Our results show that training with material-specific data yields significantly better performance than training without it. These gains cannot be replicated through synthetic noise injection, which highlights the importance of training with material data. Notably, under our experimental conditions, models trained on data from 14 materials, selected using quota sampling based on their $T_c$ values, can match the performance of models trained on all 73 materials. Overall, our results show that material-property guided data selection reduces training-data requirements, supporting scalable deployment of radar-based gesture recognition systems.

## Background and related work

### Radar sensing technologies

Radar sensing uses radio-frequency (RF) waves to detect motion by transmitting pulses via a Tx antenna and analysing the echoes received by an Rx antenna to extract object properties such as velocity and radar cross-section^9^. Unlike RGB camera or infrared (IR) based systems, radar is unaffected by lighting conditions and can penetrate non-metallic materials, making it suitable for sensing through occlusions. It has thus been applied in gait analysis^10,11^, movement recognition^10,12–14^, sleep and respiration monitoring^15,16^, and gesture-based interaction^14,17–23^, across frequencies from 2.4 GHz^16,23^ to 24 GHz^15,19^.

Millimetre-wave radars operating at 50–70 GHz offer high spatial resolution, making them ideal for detecting fine-grained finger movements and complex hand gestures^24,25^. Their compact size, low power consumption, and scalable manufacturing further support integration into miniaturised devices^24^. These sensors can capture subtle, non-rigid 3D gestures, including overlapping finger motions, with high precision^26^.

Prior work has demonstrated effective gesture recognition for micro-gestures^26,27^, object manipulation^25^, hybrid gesture interaction^28^, and applications ranging from augmented reality^29^ to musical expression^30,31^. Some studies have also shown that mmWave radars can differentiate materials placed directly over them^32,33^. While these studies often assume clear line-of-sight or minimal occlusion, real-world embedded applications require robust recognition through diverse materials. However, the impact of sensing *through* everyday materials on gesture recognition performance has not been systematically investigated. This paper addresses this gap by examining how occluding materials affect mmWave radar gesture recognition and by proposing strategies for selecting a minimal set of training materials to reduce data collection while preserving accuracy.

### Sensing through materials

When sensing through materials, the radar signal must propagate through an occluding layer before capturing motion. While technologies like acoustic and capacitive sensors support such interactions^34,35^, radar offers distinct advantages, including higher spatial resolution^36^, touchless interaction (unlike capacitive sensors), and longer-range detection (unlike acoustic sensors). The emergence of low-cost on-chip sensors such as Google Soli,^1^ and UltraSense TouchPoint,^2^ together with low-power embedded microcontrollers such as TI’s MSP430^3^ and advancements in deep learning have enabled rich gesture interactions^27,37^, often outperforming acoustic^38–42^ and capacitive approaches^43–45^. Despite these advances, only a few studies systematically explore radar-based gesture sensing through materials.

Existing work on RF-based through-material sensing focuses largely on detecting poses, objects and people behind walls^46–50^ or common materials^22,51^, and coarse activity recognition on surfaces^52,53^. Some efforts aim to correct signal distortions via denoising autoencoders^54–56^ and Convolutional Neural Networks (CNNs) to counteract radar interference^57–59^. However, fine-grained gesture recognition through materials remains under explored. Only a few studies have attempted this^3,4,51^, and none systematically use material-specific training data, which our work addresses.

### The effect of materials on radar signal quality

When sensing through materials, the radar signal must propagate through an occluding material twice. Since different materials have different characteristics, the signal will be affected in different ways. The effect can range from signal attenuation (low energy)^60^ to signal distortion (shape change)^61,62.^ These changes can affect gesture recognition performance.

Čopič Pucihar et al^3^ investigated how to characterise materials with respect to radar-based gesture recognition performance and, in doing so, addressed the attenuation-versus-distortion question. They quantitatively compared several candidate descriptors (e.g., transmission coefficient $T_c$, SNR, received energy, and amplitude) and found that Tc (capturing attenuation) is the strongest predictor of recognition performance degradation, substantially outperforming SNR (Spearman’s *ρ = 0.86* for Transmission Coefficient vs. *ρ = 0.62* for SNR).

Moreover, obtaining reliable, repeatable measurements of signal distortion characteristics for FMCW mmWave radars, such as phase/frequency-dependent effects across the sensor bandwidth (57–64 GHz), typically requires specialised instrumentation (high-frequency network vector analysers) and calibration procedures, which are expensive and not widely available. Building a training method that depends on such hard-to-acquire measurements would substantially limit the practical applicability of the approach in real-world deployments.

Based on the above reasons, we selected $T_c$ as a practical proxy for material-induced sensing degradation guiding the material selection and training design.

### Material selection

Since training a gesture classifier using every possible material is impractical, researchers typically rely on sampling or optimisation methods to choose a representative set. The effectiveness and generalisability of the model depend on how well this subset reflects the broader material population.

#### Sampling techniques

We studied two sampling approaches to select an optimal set of materials per sampling approach for training gesture recognisers: Systematic (or Random) and Quota sampling. *Systematic sampling* is a probabilistic technique that ensures a unbiased, representative selection of a larger population^63^ and is valued for its simplicity and effectiveness^63,64^. *Quota sampling,* by contrast, is a non-probabilistic technique that enables more targeted selection based on specific characteristics^65^, such as $T_c$ in our case. By clustering materials according to their $T_c$ and selecting samples from these clusters (e.g., choosing the centroid or the nearest material), Quota sampling ensures that our subset of materials covers the full spectrum of $T_c$ values in the training dataset.

#### Optimisation algorithms

Optimisation algorithms are essential for solving problems where an objective function can be defined. Some of these algorithms include Grid Search^66^, Random Search^67^, Genetic Algorithms (GAs)^68^, Tree-structured Parzen Estimator (TPE)^69^, and Bayesian Optimisation (BO)^12,68,70,71^.

A comparative study by Liashchynskyi et al^72^ indicated that GAs often outperform Grid and Random Search in terms of efficiency and effectiveness. Moreover, BO has been noted for its superior performance over GAs^73^ and TPE^74^ due to its ability to efficiently explore and exploit the search space^12^, particularly under limited resources or high-dimensional parameter spaces^75,76^. We therefore use BO as a strong optimisation-based baseline for material selection in our study (hereafter referred to as Bayesian optimisation sampling), and we use it to sample materials and compare its performance against the two sampling techniques described above.

**Fig. 2:**
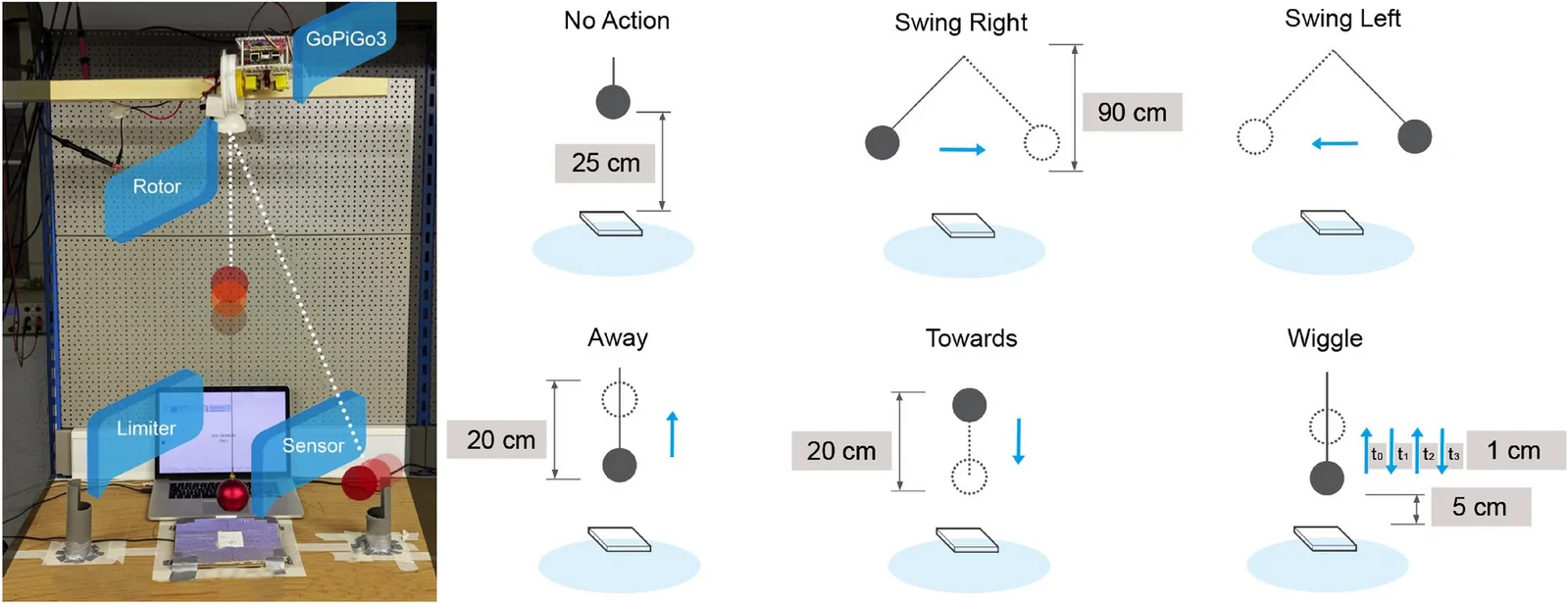
Experimental setup and the gesture set. Left figure shows the experiment setup and the right figure shows the executed gestures with the robot. On the right figure filled and dashed circles denote the initial and final position of the ball respectively. The same gesture set was used as in^3^.

## Experiments

We chose six distinct gestures (Fig. 2) informed by a survey of previous studies using mmWave radar sensing for interaction^3,27^. Further, since it is crucial to execute gestures with minimal articulation variation, we replicated the mechanical system proposed in prior work^3^.

We experimented with two different deep learning architectures: a 3D convolutional neural network (referred to as *Conv3D*) and a 2D convolutional neural network using spectrograms (referred to as *Spectrogram*) derived from previous work^3^ with one key difference when preprocessing data. Here, Range Doppler^36^ images from 4 Rx antennas are not averaged, but concatenated into one sequence. Refer to *Section *1 of Supplementary Materials for more details about architectures.

We trained all classifiers with the Adam optimiser with learning rate *η =0.001* and decay rates $\beta _1=0.9, \beta _2=0.999$. We set the maximum number of epochs to 400, and also set an early stopping criteria of 100 epochs, which means that training stops if the validation loss does not improve after 100 epochs, and the best classifier weights are retained.

The early stopping threshold was set to a high number of epochs due to the grokking phenomenon observed during training^77^, where the model initially overfited, showing a large gap between training and validation accuracy, before eventually converging to near-perfect performance on both. This transition from overfitting to generalisation required extended training time.

### Data collection

We collected the radar signals from 40 repetitions of six gestures sensed through 73 everyday materials^3^, resulting in a total of 17,520 recordings. The materials used were identical to those reported in the material catalogue^4^by Čopič Pucihar et al^3^. We primarily focused on non-conductive materials such as fabrics, plastics, and wood, as radar waves do not effectively penetrate conductive materials like metals. For instance, the KAPA plate, which contains a thin aluminium layer $\tilde{0}$, exhibits a transmission coefficient of just 0.14, indicating that 86% of the signal amplitude is attenuated. Such materials are therefore unsuitable for radar sensing applications and were not included in material training. Additionally, we recorded 200 repetitions of each gesture without any material occluding the sensor, totalling 1,200 recordings.

Previous research has demonstrated that Range Doppler^36^ images can be used for robust gesture recognition^4,23,78^, so we opted to use them in this experiment as well. Each gesture is represented as a sequence of Range Doppler images, where each image depicts the reflected energy intensity as a function of target range (radial distance from the radar) and velocity (direction and magnitude of the target). Positive velocity corresponds to motion away from the radar (increasing range), while negative velocity indicates motion towards the radar (decreasing range). One Range Doppler image was computed for each transmitter-receiver antenna pair.

We used the Google Soli sensor, a Frequency-Modulated Continuous-Wave (FMCW) radar operating in the 57–64 GHz range. The sensor was configured following our prior work^2^, with a sampling rate of 1000 Hz, Doppler range sensitivity set to [−16, 0], and the built-in adaptive clutter filter disabled. Experiments were conducted using Soli’s default mode, which activates one transmit (Tx) and four receive (Rx) antennas.

### Statistical analysis

We first consider parametric statistical tests (e.g., paired t-tests, repeated-measure ANOVA) and verify their assumptions by testing normality with the Shapiro–Wilk test and homogeneity of variance with Levene’s test. When assumptions are violated, we use non-parametric alternatives, such as the Friedman test followed by post-hoc Wilcoxon signed-rank tests. We apply Bonferroni correction to control for multiple comparisons. For pairwise comparisons, we use two-tailed tests by default, and one-tailed tests only when the hypothesis is directional. In particular, one-tailed tests are used in subsection Selecting the optimal number of materials (RQ4), where we estimate the optimal number of training materials using a significance-based procedure.

### Data partitioning

We aimed to evaluate several training strategies, including how incorporating material-specific data affects classification performance. To ensure that the effects observed were due to the introduction of material-specific data, it was crucial to control for other confounding variables, such as the size of the training partition. Therefore, we needed a sophisticated data partitioning method that introduced material-specific data without increasing the size of the training partitions. This was achieved by reducing the number of repetitions for each material.

To accomplish this, we first limited the data partition size to 630 repetitions across the six gestures. Given the amount of data available for each material, this was the maximum partition size that allowed us to build a classifier using a minimum of three materials. We consider this size appropriate because it is comparable to the 840 repetitions used in previous work for training and validating classifiers with similar architectures and gesture sets^2,3^. This decision was further supported by pilot experiments, where classifiers trained and evaluated with 840 and 630 repetitions, respectively, showed comparable performance levels despite the difference in partition size.

**Fig. 3:**
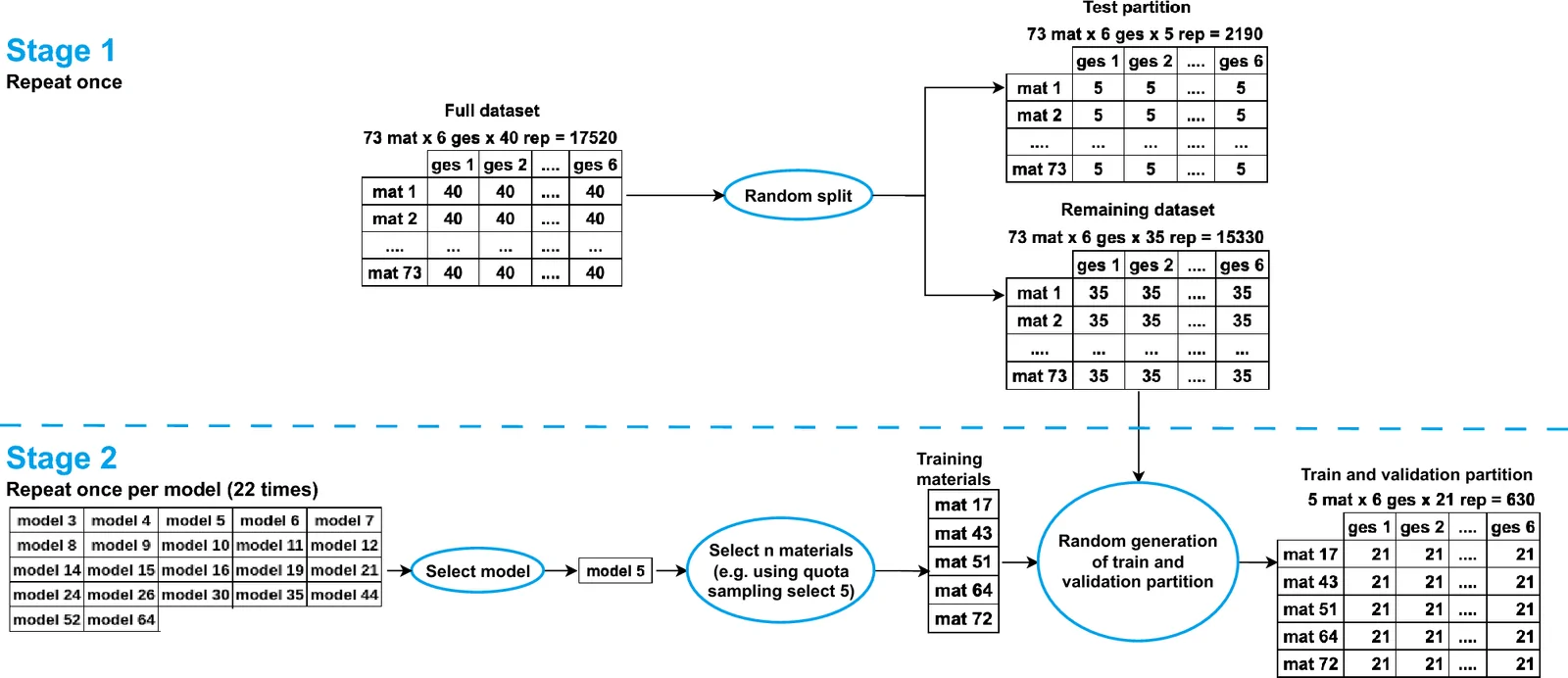
Data Split. In Stage 1, the entire dataset (73 materials *×* 6 gestures *×* 40 repetitions) was randomly divided to create a test partition, which consisted of 73 materials *×* 6 gestures *×* 5 repetitions. All trained classifiers were evaluated on this unseen test partition. In Stage 2, the remaining data was used to generate training and validation partitions. These partitions were created randomly, but the specific configuration depended on the number of training materials and the method used for material selection. For example, the figure illustrates partitioning for a classifier trained with 5 training materials selected using *Quota sampling*. Despite varying the number of training materials, the sizes of the training and validation partitions remained consistent. This was achieved by adjusting the number of repetitions per gesture we include in each partition.

The data partitioning process was conducted in two stages. In Stage 1, we split the data to create the test partition. In Stage 2, the remaining data was used to generate training and validation partitions. These partitions were created randomly, but the specific configuration depended on the number of training materials and the method used for material selection. Despite varying the number of training materials, the sizes of the training and validation partitions remained constant. This was achieved by adjusting the number of repetitions per gesture included in each partition. This process is illustrated in detail in Fig. 3.

### Training with synthetically generated noise (RQ1 & RQ2)

To determine whether training with materials improves performance (**RQ1**) and whether similar performance improvements can be achieved through synthetic noise^79^ generation (**RQ2**), we trained and compared the classifiers under six conditions:

- *No-material* classifier trained only on data where the sensor was not occluded by materials (i.e., *No-material dataset*).
- *All-materials* classifier trained with all 73 materials (i.e., *Material dataset*).
- *Gaussian-noise* classifier, where Gaussian noise was added to the training partition (i.e., *No-material dataset* + *Gaussian-noise*).
- *RandAugment* classifier where training partition was modified using stochastic data augmentation routine designed specifically for image-classification problems (i.e., *No-material dataset* + *RandAugment*).
- *RandAugment with Gaussian-noise* classifier, where training partition was first transformed with Gaussian-noise and then augmented through stochastic data augmentation (i.e., *No-material dataset* + *RandAugment with Gaussian-noise*).
- *CCycleGAN* classifier, where training partition was first transformed with Conditional CycleGAN we specifically trained for this purpose (i.e., *No-material dataset* + *CCycleGAN*).

The partition sizes were kept consistent throughout all conditions and the aforementioned training procedures were followed. To make our evaluation more robust, we retrained our classifiers three times, each time generating new data partitions according to a different random seed. We report average classification performance^3^, a combination of AUC (Area Under the ROC Curve) and Weighted Accuracy, defined as $\text {AvgPerf} = (\text {Accuracy} + \text {AUC}) / 2$. This combined metric captures both how often predictions are correct and how well the model’s confidence scores distinguish the correct gesture from the others. Both Accuracy and AUC scores are weighted by class support. In the supplementary materials (Section 6) we additionally report the individual Accuracy, AUC, and Micro-F1, to provide further insights into the performance results. Details regarding synthetic noise generation are provided in the next section.

**Fig. 4:**
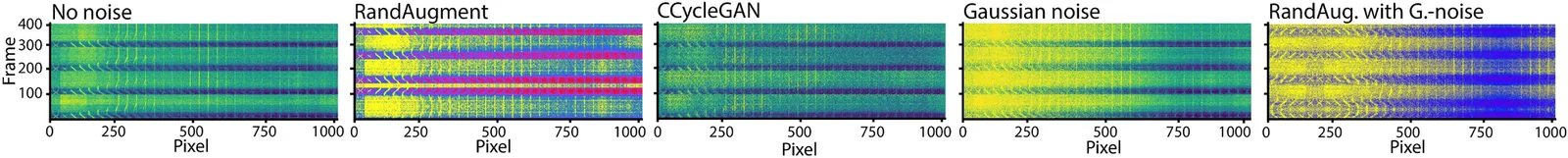
Spectrogram images with injected synthetic noise using different methods. The x-axis represents 1024 px which are flattened from a *32 × 32* Range Doppler image. The y-axis represents frames as a time series: We stack 4 time series images on top of each other, each representing one receiving antenna.

#### Synthetic noise generation

All data augmentations in this study were performed on spectrogram images, created by flattening *32 × 32* Range Doppler images into vectors of 1,024 values and stacking them, which resulted in a *400 × 1024* px image (Fig. 4 top-left). For the *Gaussian-noise* classifier, we generated Gaussian noise with a mean of 0.001 and a randomly selected noise level, choosing a standard deviation within the [0,1] range. This approach simulated varying noise level for materials with different $T_c$ values (i.e., the lower the $T_c$, the higher the noise level and vice versa). Next, we applied a linear gradient mask starting at a random pixel value and ending at the 512^th^ pixel (to target the approximate volume around the sensor where gestures were performed). Finally, the generated noise was added to the spectrogram image, and all values were clipped to the [0,1] range. An example of a modified image can be see in Fig. 4.

When preparing data for the *RandAugment* classifier, we used stochastic data augmentations procedure^80^: value range bounds were set between 0 and 1, with 5 augmentations per image and a magnitude of 0.5^80^. We considered 10 transformations: AutoContrast, Equalize, Solarize, Color, Contrast, Brightness, ShearX, ShearY, TranslateX and TranslateY. An example of a modified image can be seen in Fig. 4.

For the *RandAugment with Gaussian-noise* classifier, we first applied Gaussian noise to the image and then used RandAugment on the noisy image (seen Fig. 4). The procedures for both steps followed the methods described earlier.

Finally, for the *CCycleGAN* classifier, we trained a Conditional CycleGAN (CCycleGAN)^81^ to convert *No-material dataset* samples to *Material dataset* samples. CCycleGAN is an extension of the popular CycleGAN^82^ architecture, where the generation process is conditioned on class labels. Specifically, we introduced as labels the gesture type and the material type to the learned (image-based) latent space, so that the decoder could generate spectrograms of specific gestures performed either with or without materials, using unpaired input and output images. The model hyperparameters were kept consistent with those outlined in the original CCycleGAN paper^81^ to ensure a fair alignment with established benchmarks. An example of a modified image can be seen in Fig. 4.

#### Results

**Fig. 5:**
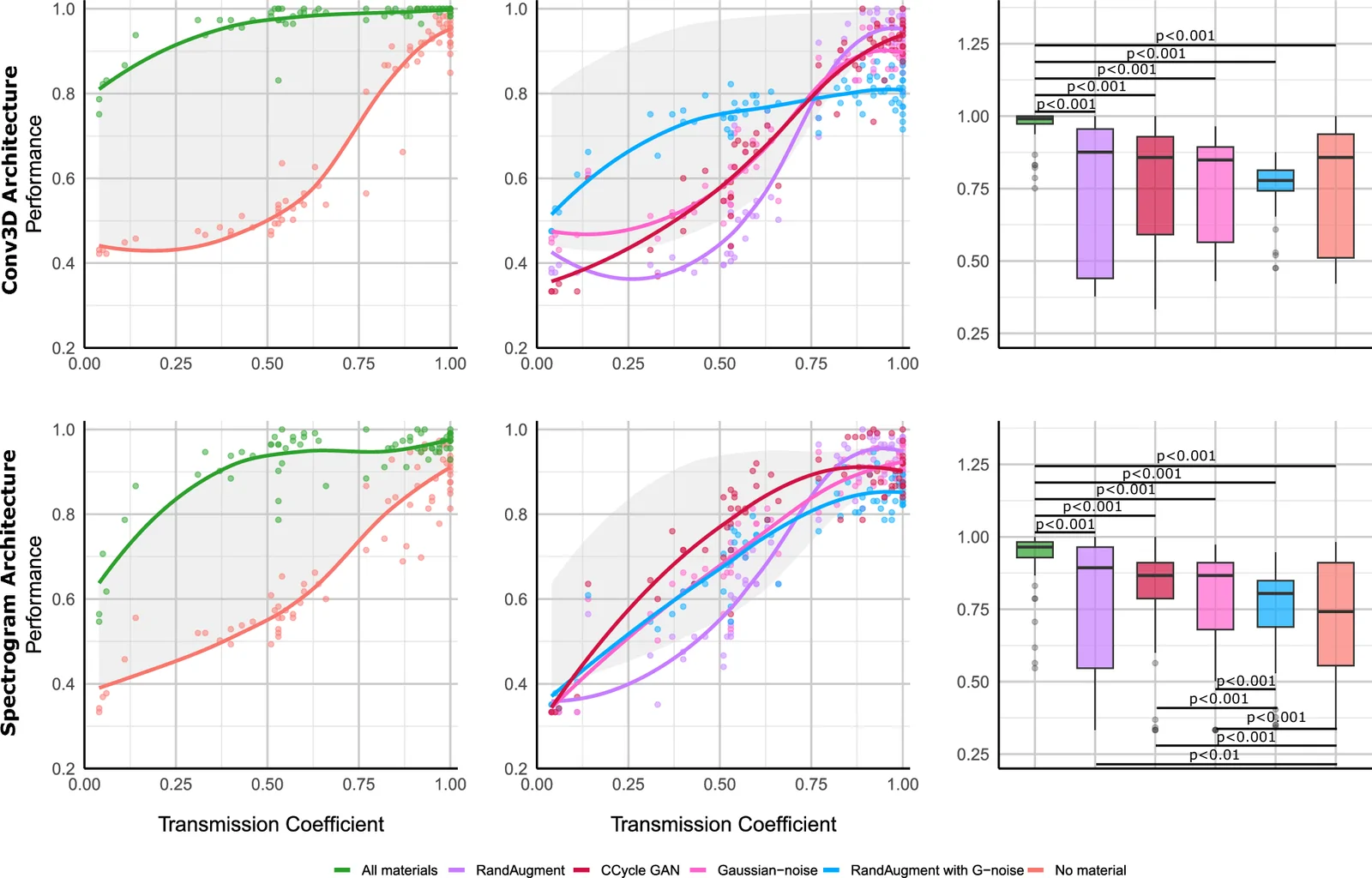
Comparing classification performance when training with synthetically generated noise. We compare *All-materials*, *No-material*, *Gaussian-noise*, *RandAugment*, *RandAugment with Gaussian-noise* and *CCycleGAN* classifiers for Conv3D (top) and Spectrogram (bottom) architectures. For Friedman and Wilcoxon signed-rank post-hoc statistics refer to *Section* 3 of Supplementary Materials.

The results for the Conv3D architecture demonstrate that the *All-materials* classifier achieves the best classification performance across the entire range of $T_c$ (see the green line in Fig. 5 top-left). For the Spectrogram architecture, the *All-materials* classifier performs best over most of the $T_c$ range (see the green line in Fig. 5 bottom-left).

The box plots on the right of Fig. 5 illustrate the classifier performance for both Conv3D and Spectrogram architectures, including: *No-material*, *All-materials*, *Gaussian-noise*, *RandAugment*, *RandAugment with Gaussian-noise* and *CCycleGAN* classifiers. We performed normality and equal variance assumption analyses and identified violations of these assumptions (see *Section* 2 in Supplementary Materials for details). Consequently, we conducted non-parametric tests, including the Friedman test and post-hoc Wilcoxon signed-rank tests with Bonferroni-corrected p-values. The results of these tests indicate that the *All-materials* classifier significantly outperformed all other classifiers for both architectures (Fig. 5 right), suggesting that synthetic data augmentation did not achieve the performance levels of a classifier trained with noise induced by real materials.

Furthermore, in the Conv3D architecture, post-hoc tests revealed that none of the classifiers trained with synthetically augmented data outperformed the *No-material* classifier. In contrast, for the Spectrogram architecture, synthetic data augmentation led to significant performance improvements. The *Gaussian noise* classifier (*W = 320, r = -0.73, p < .001*), *RandAugment* classifier (*W = 529, r = -0.55, p < .01*), and the *CCycleGAN* classifier (*W = 456, r = -0.65, p < .001*) all showed significant improvements over the *No-material* classifier.

These results show that diversifying our data by training with material-specific data improves classification performance, making our classifiers more robust to material occlusion. However, it also indicates that the tested augmentation methods failed to approximate the performance of a classifier trained with materials, especially when aiming to approach the performance of the All-materials classifier. However, training with material-specific data requires a lot of resources and effort for acquiring the data and retraining classifiers (i) every time the gesture set changes, or (ii) when we want to sense though a new set of materials. Therefore, in the following section, we focus on optimising this process by minimising the amount of material-specific data required while trying to match the performance of our best classifier (i.e., *All-materials* classifier).

### Analysis of sampling techniques (RQ3)

We experimented with three techniques defined in subsubsection Sampling techniques: *Systematic*, *Quota*, and *Bayesian* optimisation sampling. All 73 materials were characterised based on their $T_c$values reported in the materials catalogue published by Čopič Pucihar et al^3^.

#### Systematic random sampling

All materials were first sorted in ascending order according to their $T_c$ values. Next, we randomly selected the starting point and then every *n*^th^ element, where *n* depended on the number of materials we needed to sample from the set. For example, to select 100 elements from a population of 1000, we would randomly choose a starting point in the range between 1 and 10 and then select every 10^th^ element. In our case, this selection process was repeated 22 times for each unique number of materials we experimented with.

#### Quota sampling

We selected materials based on a budget. For example, to select 100 elements from a population of 1000, we would divide the population into 100 element categories using clustering and then select one element from each category. In our study, we applied K-means clustering to group the 73 materials based on their $T_c$ values into *n* clusters, where *n* corresponded to the number of materials we needed to sample. Once the clusters were formed, we selected the material closest to the centroid of each cluster. This selection process was repeated 22 times, once for each unique number of materials we experimented with.

#### Bayesian optimisation sampling

We treated material selection as an optimisation problem. We used $T_c$ values to define the search space, aiming to identify a set of training materials with $T_c$ values that would maximise classification performance. We implemented BO with the GPyOpt package^83^, adhering to its default hyperparameters. Due to the high computational cost (i.e., *∼ 45* minutes per Conv3D classifier on Quadro RTX 6000 GPU) we limited, based on the literature, the maximum number of iterations to 15. Because Bayesian optimisation requires repeated model training and evaluation, as well as access to a full material dataset, it is computationally expensive and complicates practical deployment. We therefore treat it as a theoretical upper bound rather than a recommended practical strategy.

Bayesian optimisation is known for its effectiveness in converging to near-optimal solutions with appropriately chosen priors and acquisition functions. For example, Snoek et al^71^. demonstrated that the Branin-Hoo function, a standard benchmark in Bayesian optimisation, achieved near-optimal convergence within the first 15 iterations. They also observed rapid convergence when tuning nine hyperparameters of a convolutional neural network (CNN). While Wang et al^84^ also reported near-optimal solutions for a body part classifier within 15–20 iterations. Our experiments also showed that performance typically plateaued at around 15 iterations, thus we chose this hyperparameter.

#### Evaluation method

Each sampling technique was evaluated by training 22 classifiers that varied in the number of materials used for training. The choice of materials varied depending on the sampling technique. We maintained a consistent data partition size and used the same test partition for all experiments, as detailed in Fig. 3. To ensure robustness in our evaluation, we retrained each classifier three times, generating new data partitions for each run. For each iteration, we recorded the classification performance, defined as $\text {Performance} = (\text {Accuracy} + \text {AUC}) / 2$, across the 73 materials. We conducted normality and equal variance assumption analyses and identified assumption violations (see *Section* 2 in Supplementary Materials for details). Consequently, we assessed differences among the sampling techniques using non-parametric tests, including the Friedman test and post-hoc Wilcoxon signed-rank tests, with Bonferroni-corrected p-values.

#### Results

**Fig. 6:**
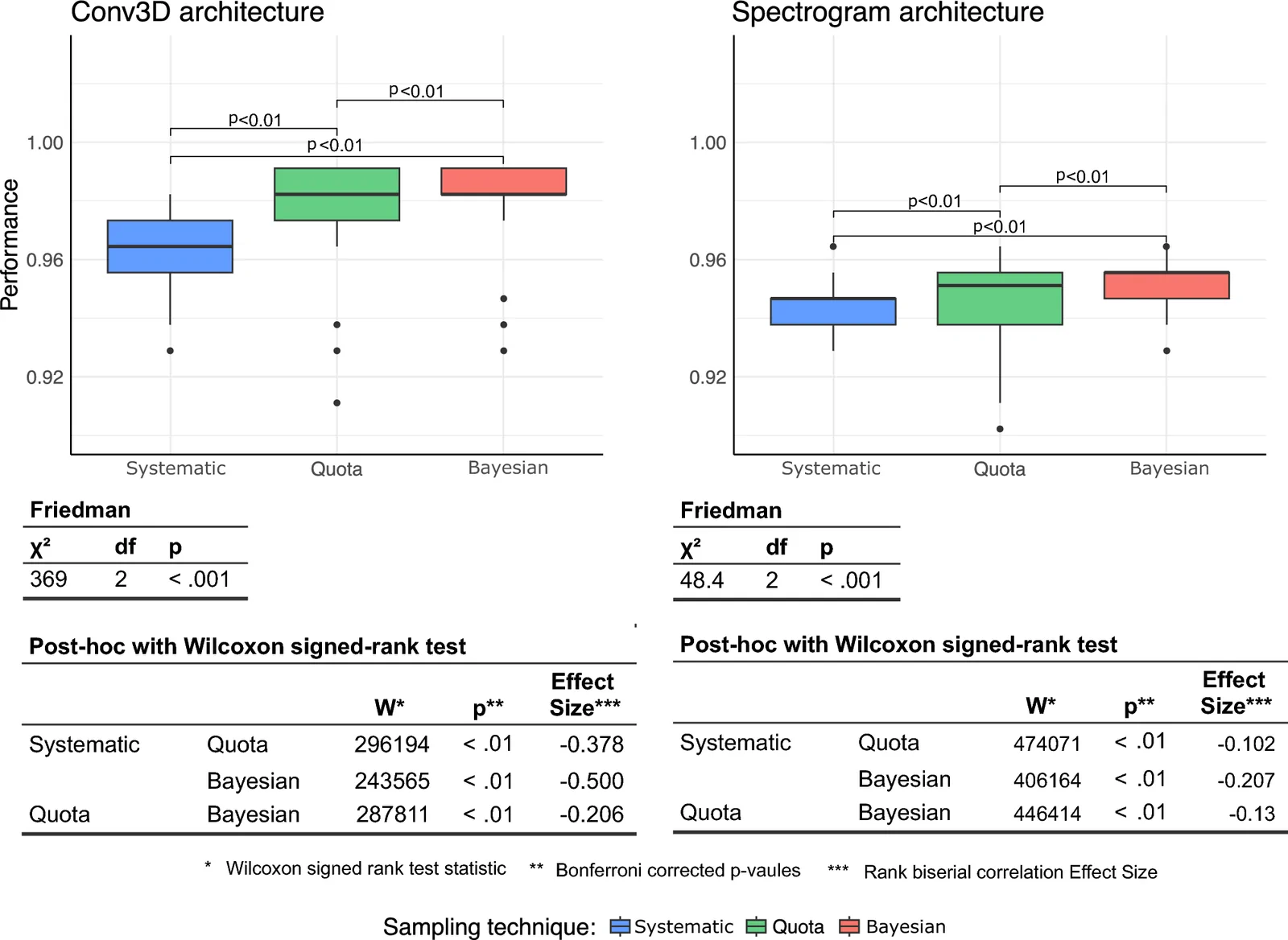
Comparing classification performance using *Systematic*, *Quota*, and *Bayesian* optimisation sampling for both Conv3D and Spectrogram architectures. Each distribution is represented by the average performance scores of 22 classifiers across 73 materials, totalling 1606 data points. The top graphs display box plots of performance, while the bottom tables present Friedman statistics and post-hoc Wilcoxon signed-rank test results.

In Fig. 6 we present the overall classification performance of classifiers trained with a reduced set of materials (e.g., 3, 4, 5, 6,..., 64), with material selection done using *Systematic*, *Quota*, and *Bayesian optimisation sampling* for both Conv3D and Spectrogram architectures. The results show noticeable differences in classification performance between the sampling techniques (see Fig. 6 top graphs). The Friedman test revealed significant differences for both Conv3D ($\chi ^2(2)=369$, *p<.001*) and Spectrogram ($\chi ^2(2)=48.4$, *p<.001*) architectures. Subsequent pairwise comparisons indicated that *Bayesian optimisation sampling* consistently outperformed the other techniques, while *Systematic sampling* performed the worst. This trend was observed for both architectures (Fig. 6 bottom tables), although the effect sizes were smaller for the Spectrogram architecture, suggesting less pronounced differences. Furthermore, the effect sizes for the differences between *Bayesian optimisation* and *Quota sampling* were also relatively small (*r=-0.13* and *r=-0.206* for Conv3D and Spectrogram respectively), indicating smaller differences compared to other sampling pairs.

### Selecting the optimal number of materials (RQ4)

We anticipated that different sampling techniques would exhibit varying saturation points, with higher-quality sampling methods leading to faster saturation and superior overall performance. Thus, we started our experimentation by identifying saturation points for different sampling techniques. Next, we sought to better understand which materials were selected and whether there exists a set of influential materials that best improves the performance of the classifier.

#### Objective functions

We considered two objective functions: *Significance-based* and *Multiple-criteria-based*. In the *Significance-based* function the optimal number of training materials is the smallest number at which no significant difference in performance exists between the selected classifier and the *All-materials* classifier trained with all materials (i.e., *All-materials* classifier). The only downside of this approach is that it relies on a single parameter. In the *Multiple-criteria-based* function the optimal number of training materials is the number of materials included in the best-performing classifier, considering four selection parameters: (1) median performance (higher is better); (2) variance of performance (lower is better); (3) number of materials (lower is better); and (4) significance level *p* (higher is better).

#### Evaluation method

To determine the optimal number of materials according to *Significance-based* objective function, we first conducted assumption tests to select the appropriate statistical methods. We then performed a single-tailed test, adjusted with Bonferroni correction for multiple comparisons, to compare the classification performance of the *All-materials* classifier with 22 classifiers trained on reduced sets of materials (*N-materials*, where *N=3,4,5,6,...,64*). This test reveals whether the *All-materials* classifier significantly outperforms the *N-materials* classifiers, where the alternative hypothesis is $H_a: \text {diff}> 0$, with $\text {diff} = \text {Perf}_\text {All-materials} - \text {Perf}_\text {N-Materials}$. As soon as this is not the case, there is no performance gain from adding more material-specific data, and we can assume that the saturation point was reached.

To determine the optimal number of materials according to *Multiple-criteria-based* objective function, we used a Multi-Criteria Decision-Making (MCDM) algorithm. Among various MCDM techniques we chose the Technique for Order Preference by Similarity to Ideal Solution (TOPSIS), since it is a widely used method^85^ that is transparent and easy to understand^86^. In this context, the ideal solution would involve the fewest materials (e.g., 3), the highest median performance (e.g., 100%), and the minimum variance in performance (e.g., 0). Conversely, the negative ideal solution would consist of the maximum number of materials (e.g., 64), the lowest performance, and the highest variance (e.g., 1).

#### Results: Optimal number of materials

**Fig. 7:**
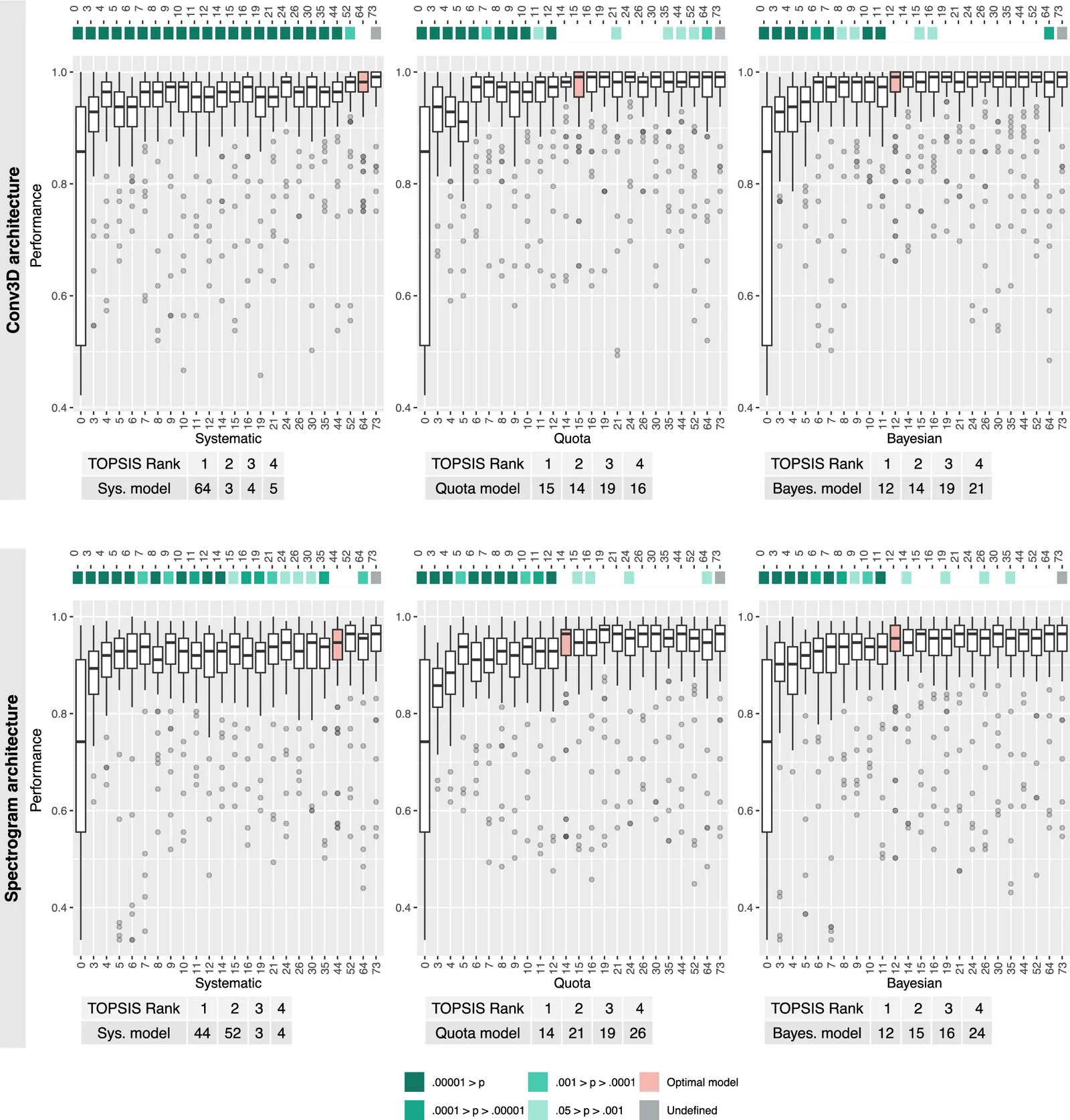
Change of gesture-classification performance as the number of training materials increases. Columns compare the three material-selection methods (*Systematic sampling*, *Quota sampling*, *Bayesian optimisation sampling*) and rows compare the two model types (Conv3D, Spectrogram). At the top part of each figure, we present the results of a Wilcoxon signed-rank one-tailed test comparing the *All-materials* classifier (trained with 73 materials) against classifiers trained with reduced sets of materials (N-materials: *N = 3, 4, 5, … , 72*). This test evaluates whether the reduced-material model performs significantly worse. The orange-highlighted point marks the smallest number of materials whose performance is not significantly worse than the 73-material baseline (our selected “best practical” model). The table below each plot lists the TOPSIS ranks of the four best reduced-material models to show which selections perform best.

The results in Fig. 7 illustrate how classification performance evolves with an increasing number of materials for various sampling techniques and classifier architectures (Conv3D and Spectrogram). These results validate our assumption that classification performance improves with an increasing number of training materials, but eventually plateaus as the classifier saturates. Additionally, we observe a reduction in performance variance as the number of materials increases. Notably, the *Bayesian optimisation sampling* (Fig. 7 right column) shows a smoother performance increase compared to *Systematic* and *Quota* sampling (Fig. 7 left and middle columns, respectively). This trend is consistent across both Conv3D and Spectrogram architectures.

At the top of each plot in Fig. 7 we present Bonferroni-corrected p-values from a Wilcoxon signed-rank one-tailed test. These p-values indicate whether the *All-materials* classifier significantly outperforms classifiers trained with a reduced set of materials, referred to as *N-materials* classifiers, where *N = 3, 4, 5, 6, ... 64*. Non-parametric statistics were employed due to violations of normality and equal variance assumptions (see *Section* 2 of Supplementary Materials for details).

By analysing the reported p-values, we can determine that the saturation point varies depending on the sampling technique. For example, the results of the Conv3D architecture shown in the top row of Fig. 7 reveal that in the case of *Systematic sampling*, the *All-materials* classifier significantly outperformed all classifiers until *64-material* classifier; in the case of *Quota sampling* *14-material* classifier; and in the case of *Bayesian optimisation sampling* the *12-material* classifier. Therefore, we can assume the saturation points are 64, 14, and 12 materials for *Systematic*, *Quota* and *Bayesian optimisation sampling* respectively. Similarly, we can see the saturation points for Spectrogram architecture, which are: 44, 14, and 12 for *Systematic*, *Quota,* and *Bayesian optimisation sampling* respectively. Again, the results are very similar for both architectures.

The reported p-values further highlight the superiority of *Bayesian optimisation* and *Quota* over *Systematic sampling*, as the former two techniques reached the saturation point with fewer materials. Classifiers at these saturation points can be considered optimal because they achieve the performance of the *All-materials* classifier while using the smallest possible number of training materials.

The results from the MCDM using the TOPSIS method (shown in the tables at the bottom of each plot in Fig. 7) align well with the previous significance-based selection approach. The optimal classifier, as determined by the highest TOPSIS rank, is the same in all but one case: *Quota sampling* for the Conv3D architecture (row 1, column 2 in Fig. 7). In this instance, the *15-material* classifier is identified as optimal, rather than the *14-material* classifier, which is ranked second. Despite this small discrepancy, the results are consistent overall, confirming the robustness of both the significance-based and MCDM approaches.

#### Results: Chosen materials

**Fig. 8:**
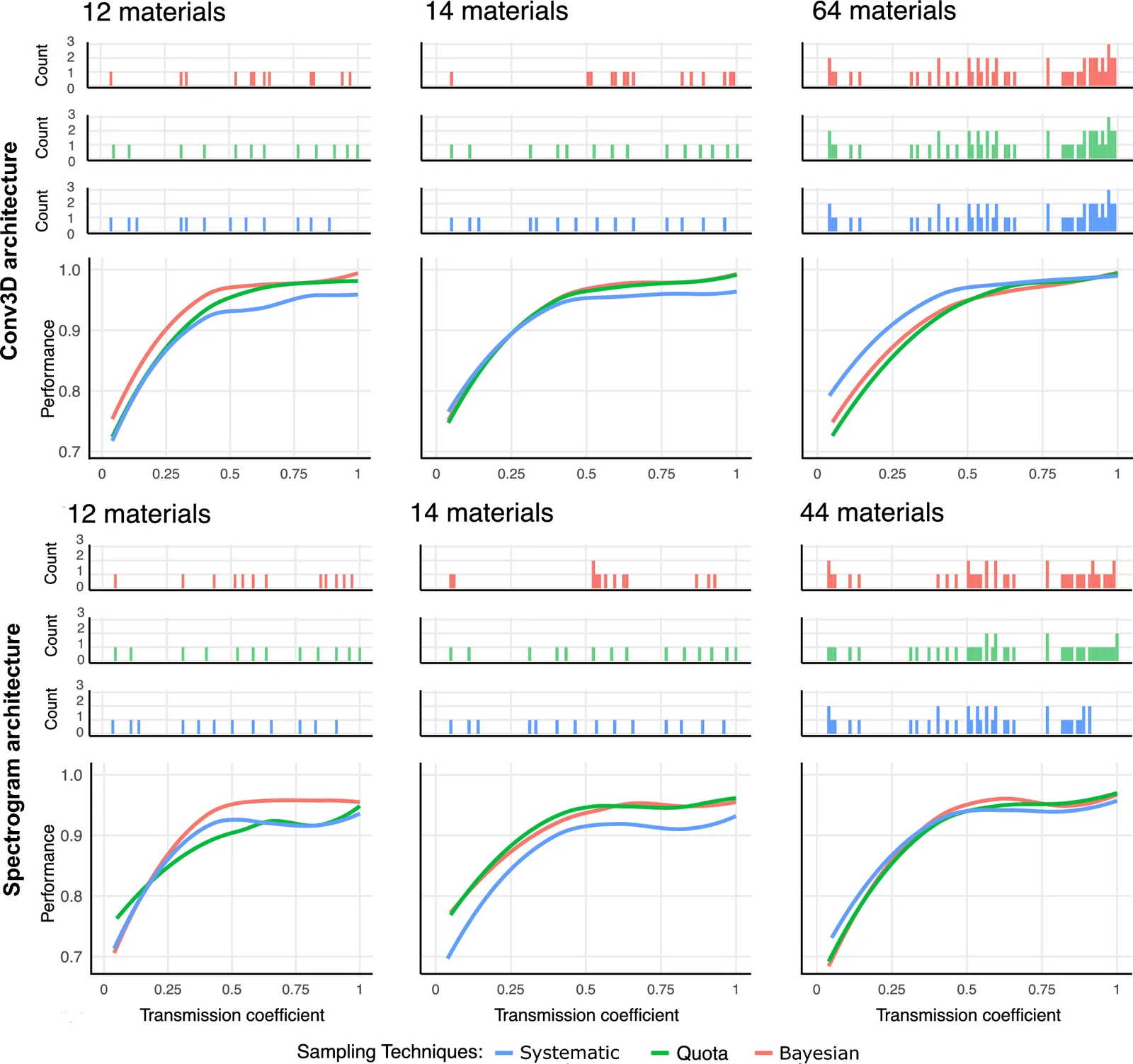
Comparison of optimal classifiers obtained using different sampling techniques for Conv3D and Spectrogram architectures. Columns show the sampling method whose optimal classifier is being highlighted (*Bayesian sampling*: left, *Quota sampling*: middle, *Systematic sampling*: right). For each column, we compare that method’s optimal classifier against classifiers trained with the same number of materials but selected using the other sampling methods (e.g., the *Bayesian 12-material* model is compared to the *Quota 12-material* and *Systematic 12-material* models). The histograms indicate which materials were selected, positioned by their transmission coefficient $T_c$. The middle plots show how classification performance varies as a function of $T_c$. Statistical comparisons (Wilcoxon signed-rank tests) and full performance distributions (box/violin plots) are provided in the supplementary material.

The results for Conv3D and Spectrogram architectures in Fig. 8 compare the optimal classifiers obtained using different sampling techniques of sampling techniques (determined in subsubsection Results: Optimal number of materials) with corresponding classifiers trained with the same number of materials but a different sampling technique. For example, *Bayesian* *12-material* classifier is considered the optimal classifier for *Bayesian optimisation sampling* and is compared with *Systematic* *12-material* classifier and *Quota* *12-material* classifier. We provide the same analysis for the Conv3D and Spectrogram architectures in *Section* 4 of Supplementary Materials with Wilcoxon signed-rank test results comparing classifier performances.

The top histograms in Fig. 8 show the distribution of selected materials across transmission coefficients ($T_c$) for various sampling techniques. *Systematic sampling* shows a notable gap in high transmission coefficient ranges, particularly in the 12-material and 14-material classifiers, indicating potential under-representation of materials with high $T_c$. Conversely, *Quota sampling* achieves a balanced selection across the full $T_c$ spectrum, ensuring comprehensive coverage and contributing to a more robust performance. *Bayesian optimisation sampling* exhibits a tendency to favour materials with higher $T_c$, which may enhance classifier performance by emphasising materials with significant influence. These patterns highlight how the choice of sampling technique affects the representation of transmission coefficients and, consequently, the overall effectiveness of the classifiers.

The middle plots of trend lines in Fig. 8 illustrate how classifier performance varies with the transmission coefficient of materials. In most cases, the optimal classifier consistently outperforms the others, except for the *Systematic 44-material* classifier with Spectrogram architecture (see *Section* 4 of Supplementary Materials). Statistical analysis, including Friedman and post-hoc Wilcoxon signed-rank tests with Bonferroni-adjusted p-values (see *Section* 4 of Supplementary Materials), confirms that the optimal classifier generally surpasses the others, except for the one mentioned above where the *Bayesian 44-material* classifier outperformed *Systematic 44-material* classifier (*W = 593, p<0.05, r = -0.412*). Additionally, for classifiers with a high number of materials (e.g., 64-material), performance variance is minimal because these classifiers include a large portion of materials, making the choice of sampling technique less critical.

## Discussion, limitations, and future work

Using a mmWave radar sensor, we collected a comprehensive dataset comprising 17,520 gesture recordings through 73 everyday materials. We evaluated several training strategies, including synthetic noise injection and three sampling techniques (systematic, quota, and Bayesian) each applied using two objective functions for determining the optimal number of training materials (i.e., the Significance-based and the Multiple-criteria-based functions). These experiments address four research questions we discuss hereafter.

### Training with real vs. synthetic material data (RQ1, RQ2)

The results presented in Fig. 5 show strong evidence that training with materials improves performance. Our *All-materials* classifier, trained on data collected through 73 different materials, significantly outperformed the *No-materials *classifier addressing RQ1. This result is also in line with preliminary work of Leiva et al^4^. However, did the classifier improve because it learnt the specific characteristics of different materials, or did it simply become more resilient to noise by being exposed to noisy data? If the latter is true, we should be able to observe similar improvements using synthetic data augmentation. The results shown in Fig. 5 indicate that this approach falls short, as none of the data augmentation techniques tested (i.e., *Gaussian-noise*, *RandAugment*, *RandAugment with Gaussian-noise*, or *CCycleGAN*) could match the performance of the *All-materials* classifier, thereby answering our RQ2. However, training with materials is resource-intensive, as it requires collecting data through each material. This highlights the need to optimise the training process, particularly in determining which materials to select and how many, as discussed next.

### Which materials to select (RQ3)?

The results presented in Fig. 6 highlight that different sampling techniques achieve different results; however, we were unable to identify a specific influential set of materials that would improve the classifier performance (Fig. 8). *Bayesian optimisation sampling* was designed to iteratively search for an optimal set of materials, but it only slightly outperformed *Quota sampling*. Our analysis also reveals that *Bayesian optimisation sampling* tends to select fewer materials with low $T_c$ values, particularly when the number of training materials is limited. However, the underlying reasons for the exclusion of low $T_c$ materials remain unclear and are beyond the scope of this study. To support future research, we have released the complete list of selected materials for all experimental configurations in *Section* 5 of the Supplementary Materials.

The key takeaway is that the selection of training materials should ensure comprehensive coverage of the $T_c$ range in which the system is expected to operate. We recommend *Quota sampling* as a practical strategy because it provides broad coverage of the $T_c$ range, and, unlike *Bayesian optimisation sampling*, does not require any material-specific training data during the sampling process and can be effectively employed as long as the $T_c$ values of the encountered materials are known.

### How many materials to select (RQ4)?

The findings in Fig. 7 demonstrate a strong alignment between the *Significance-based* and *Multiple-criteria-based* objective functions. The results also confirmed our hypothesis that increasing the number of training materials enhances classification performance, but this improvement diminishes at the saturation point. Different sampling techniques reached this saturation point at different numbers of materials. *Bayesian optimisation sampling* reached saturation with 12 materials, followed by *Quota sampling* with 14 materials, and *Systematic sampling* with 44 and 64 materials for the Spectrogram and Conv3D architectures, respectively. Considering that higher-quality sampling methods will lead to faster saturation, *Bayesian optimisation* reached saturation with the fewest materials, but its advantage over *Quota* sampling was marginal. Because *Bayesian optimisation* requires repeated model training and evaluation, while *Quota* sampling provides a more practical trade-off between performance, simplicity, and deployment cost.

### Limitations and future work

Using a single scalar $T_c$ to approximate recognition degradation works best when the material mainly introduces uniform attenuation. However, it can break down for thicker barriers (20–50 mm) with a large dielectric mismatch to air (e.g., acrylic or drywall materials), because the mismatch produces multiple internal reflections in the form of internal scattering and multipath effects. These effects can raise the effective clutter level and introduce phase-dependent distortions that degrade gesture features, while the average transmission (and thus $T_c$) may remain relatively high. As a result, a $T_c$-based model can become overly optimistic for such materials. In contrast, when the dielectric mismatch is small (e.g., Styrofoam/Styrodur), interface reflections are weak and the barrier behaves closer to air, so signal structure is preserved even in thicker materials; thus. recognition performance is largely maintained even at larger thicknesses. Finally, for thin sheets that appear “highly attenuating” the $T_c$-based prediction can be too pessimistic: although some energy is lost, the barrier may introduce little reflection-driven clutter or waveform distortion, so the classifier can still reliably extract discriminative gesture signatures.

In the above scenarios $T_c$ is likely not the best descriptor of material properties. However, for material selection to be practical, we require a systematic descriptor that can be measured reliably and inexpensively in real-world settings. We therefore use the transmission coefficient $T_c$ as a proxy for overall material-induced sensing degradation: it captures the dominant through-material attenuation and, in practice, correlates strongly with recognition degradation across materials, as was shown by Čopič Pucihar et al^3^.

We do not include difference-map visualisations (i.e. subtracting the no-material range–Doppler response from the material response) in this work. While such maps can help localise attenuation or ghosting in the range and velocity dimensions, producing physically meaningful and comparable difference maps across materials is non-trivial. Simple subtraction assumes that the no-material and material measurements are perfectly aligned in time, range, and phase, and that the barrier is the only source of change. In practice, even small registration differences and barrier-induced shifts can cause subtraction to mix true attenuation with misalignment artefacts and may obscure genuine ghosting. A robust difference-map analysis would therefore require additional calibration and alignment steps and a dedicated processing pipeline, which we leave as an important direction for future work.

The main trends were observed for both deep learning architectures (i.e., Conv3D and Spectrogram), suggesting that the findings are likely generalisable to other CNN network architectures. However, in our study, we only used Google’s Soli sensor, an FMCW radar operating in the 57–64 GHz range, limiting the generalisation of the results to other FMCW sensors with similar frequency bands and front-end configuration. However, a recent literature review of 50 radar-based HCI studies found that 80% employed FMCW radars, and 63% operated within the 57–81 GHz range, suggesting that our setup is representative of the majority of current research^87^. Nonetheless, a comparative evaluation across different radar types (e.g., FMCW, CW, UWB, pulsed), frequency bands (e.g., beyond the mmWave range), and antenna configurations (e.g., varying Tx/Rx arrangements) would provide additional insights and remains an important direction for future work.

One limitation is the use of Range Doppler images as the primary signal representation method. While this method is quite common^3,26,27^, it may not be the optimal for sensing gestures through materials. Future research should explore alternative signal representation methods to determine which method offers the best performance in this context.

Another limitation is the use of a robotic system for gesture performance instead of a human hand. While this approach provides a stable dataset, it does not reflect the inherent variability of human gestures (e.g., gesture speed, articulation, hand shape, and trajectory). Consequently, our conclusions are primarily derived from a low-variance gesture execution scenario, and greater execution variability may affect the absolute performance levels. That said, prior work by Čopič Pucihar et al^3^. evaluated $T_c$-based recognition degradation on both robot-executed and human datasets and reported consistent trends between $T_c$ and performance degradation, suggesting that our main observations are likely to generalise. Nevertheless, human variability could still shift the absolute performance levels reported here, so future work should validate these findings with human-subject studies.

Gaussian noise and RandAugment are widely used data augmentation techniques that can improve classifier performance even when applied to existing datasets^80,88–90^. However, more generalisable but computationally expensive deep learning approaches exist, such as Generative Adversarial Networks (GANs) for image reconstruction and cross-domain image mapping. We experimented with CCycleGAN and observed only marginal improvements over Gaussian noise and RandAugment. In contrast, Tran et al^91^. trained a GAN on over one million real images to model the noise distribution, achieving state-of-the-art performance on real-world noisy images. The limited gains in our case likely stem from training CCycleGAN on a relatively small and less diverse radar dataset, which lacked the volume needed to model complex noise patterns effectively. This comparison underscores the importance of large-scale data and domain-specific architectures when applying generative models for realistic data augmentation.

Other advanced, computationally intensive deep learning methods for synthetic data generation include semi-supervised Variational Autoencoders (VAEs), which have been used to generate noisy data for training image restoration networks^92^, and Autoencoders (AEs) for denoising image sequences rendered via Monte Carlo methods^93^. Transformer architectures have also gained prominence for their effectiveness in sequence modelling tasks^94^, and were leveraged to design a multi-person 3D pose estimation model capable of sensing through walls by mapping camera inputs to radar data^95^. These sophisticated techniques for synthetic data generation and noise reduction present promising opportunities for advancing radar-based gesture recognition through materials.

## Conclusion

When radar sensors are embedded in everyday objects for interaction, the radar signal must pass through the occluding material twice: once during transmission and once after reflection from the performed gesture. Because of the wide variety of materials, training a general gesture classifier is costly. In this work we investigated different approaches, including synthetically generated noise and sampling techniques, for optimising the selection of training materials while maintaining high gesture classification accuracy. Under our experimental conditions, using one FMCW mmWave radar sensor, a fixed gesture set executed by a robotic system, and 73 everyday materials, models trained on 14 materials selected by quota sampling achieved accuracies of 95.8% and 91.2% for Conv3D and Spectrogram models, respectively. These results were comparable to those obtained when training on all 73 materials, which yielded accuracies of 96.8% and 91.6%. In contrast, models trained without material data performed significantly worse, with accuracies of only 66.8% and 65.8%. Data augmentation alone did not close this gap, yielding performance comparable to that of models trained without material examples.

Indeed, we have demonstrated that training with only 14 strategically selected materials using quota sampling achieves comparable performance to using the full dataset of 73 materials. Among the evaluated selection approaches (i.e. Systematic, Quota, and Bayesian optimisation sampling), Quota sampling provided the best overall trade-off between performance and practicality. Our work contributes to the field of radar sensing through materials by offering a scalable method for building efficient and cost-effective gesture recognition systems with minimal data overhead. Our code, models, and data are publicly available at https://gitlab.com/hicuplab/seeing-through.

## Supplementary Information


Supplementary Information.


## Data Availability

we openly release our complete dataset and source code. This includes over 17,000 gesture repetitions recorded through 73 materials, as well as an additional 1,200 gesture repetitions collected under no-material conditions, along with all associated training and evaluation code https://gitlab.com/hicuplab/seeing-through. In addition to the sourcecode and the data as the reviewers suggested per-condition results will be made publicly available upon publication. The dataset has approx 29 K measurements of ACC, AUC, F1 (73-materials x 22-models x 3-sampling techniques x 2-architectures x 3-repetitions). Dataset and source code available at https://gitlab.com/hicuplab/seeing-through.

## Footnotes

1 https://www.norgram.co/work/google-soli-website

2 https://ultrasensesys.com/touchpoint/

3 https://www.ti.com/microcontrollers-mcus-processors/msp430-microcontrollers/overview.html

4 https://solidsonsoli.famnit.upr.si/
