# Supplementary material for: Efficient material selection for training occluded mmWave radar-based gesture recognisers
Source: Sci Rep. 2026 Jun 13;16:26997. doi: 10.1038/s41598-026-56018-2 (PMC13522440; doi:10.1038/s41598-026-56018-2)
Supplement: Supplementary file 1 [file 41598_2026_56018_MOESM1_ESM.pdf]

Supplementary Materials

Efficient Material Selection for Training Occluded mmWave Radar-Based Gesture Recognizers

May 26, 2026

1 Classifier architectures

In this paper we experimented with two different Deep Learning architectures: a 3D convolutional neural network (referred to as *Conv3D*) and a 2D convolutional neural network using spectrograms (referred to as *Spectrogram*), both derived from previous work [1]. Hereafter we explain each architecture in detail.

1.1 Conv3D classifier

In the Conv3D architecture (Figure 1 and Figure 3 left), a sequence of Range Doppler images from 4 Rx antennas are stacked (1) and then processed with a Conv3D layer (2) that extracts feature maps followed by 3D maxpooling (3) and spatial dropout (4) layers. Layers (2) to (4) comprise blocks of 32, 64, 128, and 256 hidden units. Next, a fully connected layer creates the feature vectors, which are fed into a softmax layer (5) for class prediction ($\hat{y}$). This classifier is identical to the one proposed by Attygalle et al. [2] with one key difference when prepossessing data. Here, Range Doppler images from 4 Rx antennas are not averaged, but concatenated into one sequence. Further details regarding this architecture are available in the Supplementary Materials.

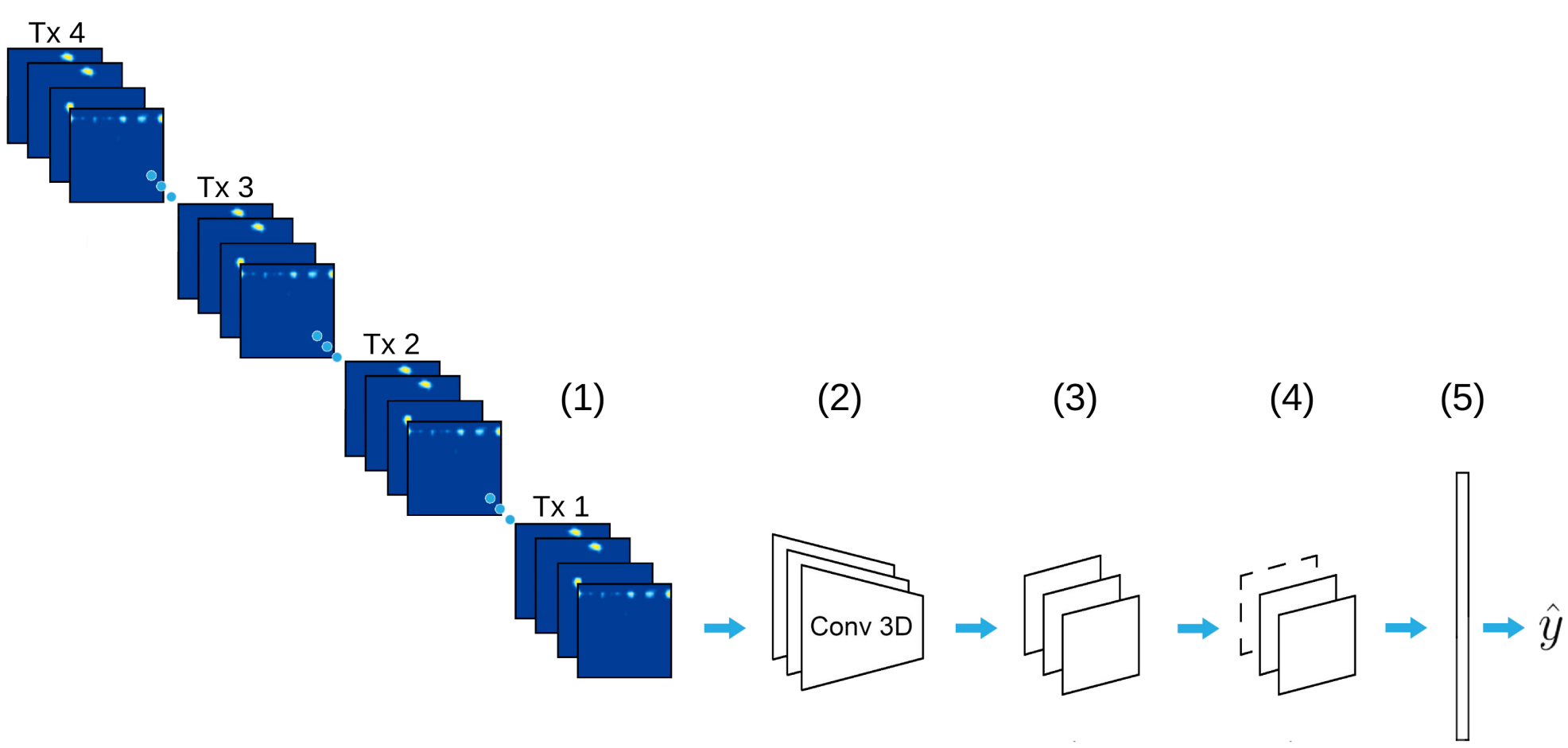

Figure 1: Conv3D deep learning classifier architecture. See Supplementary Materials for more details.

1.2 Spectrogram classifier

Convolutional-based Deep Learning architectures are common in image classification tasks. This classifier is a standard 2D CNN architecture where both the temporal and spatial information are encoded by a spectrogram image (Figure 2 and Figure 3 right). Here, a spectrogram stores information about one gesture instance, i.e., a sequence of 80×4 (antennas) 32×32 Range-Doppler images. Range-Doppler images were generated flattened into 1024 bins and stacked on top of each other, resulting in a spectrogram image of 1024×320 pixels.

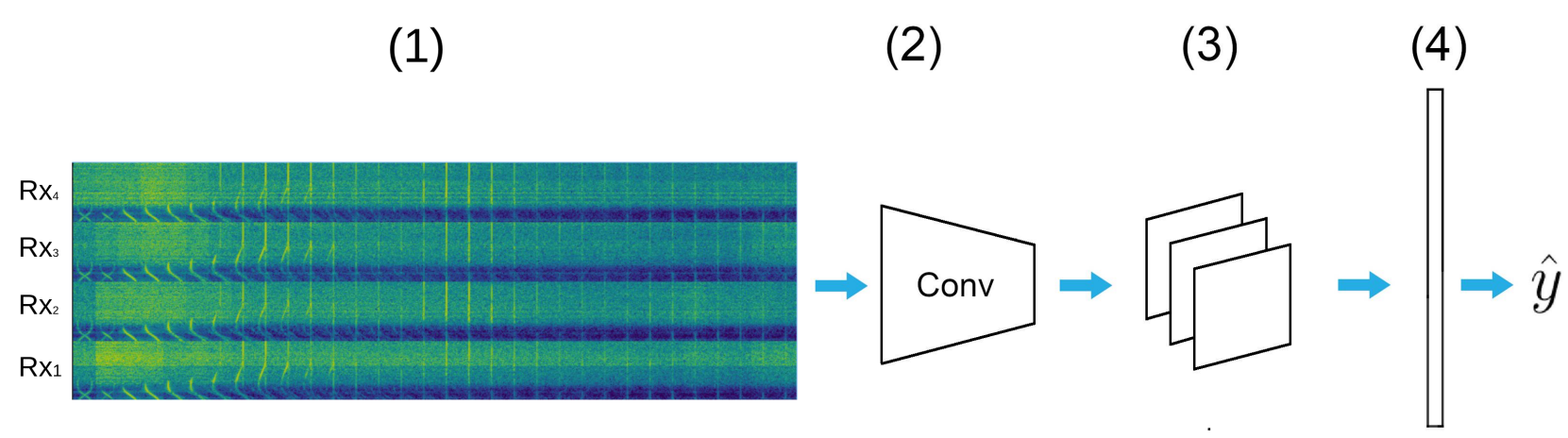

Figure 2: Spectrogram of a deep learning classifier architecture.

Each spectrogram image is processed by a stack of $32 \times 64 \times 128 \times 256$ convolutional layers with 3×3 kernels. Each CNN layer extracts feature maps from the input images and is further processed by the maxpooling layer (pool size of 2) and finally classified with a softmax layer. The classifier has 0.9M weights, which is the smallest of the classifiers considered. This is particularly desirable for applications in emended systems. This architecture is very similar to the ones used in prior work by Attygalle et al [2].

2 Normality and equal variance analysis

When selecting an appropriate statistical tool for comparing performance of classifiers trained with synthetically generated noise, we first need to check if we can use parametric statistical tools. For this purpose, we run Shapiro-Wilk normality test and Levene's homogeneity of variance test.

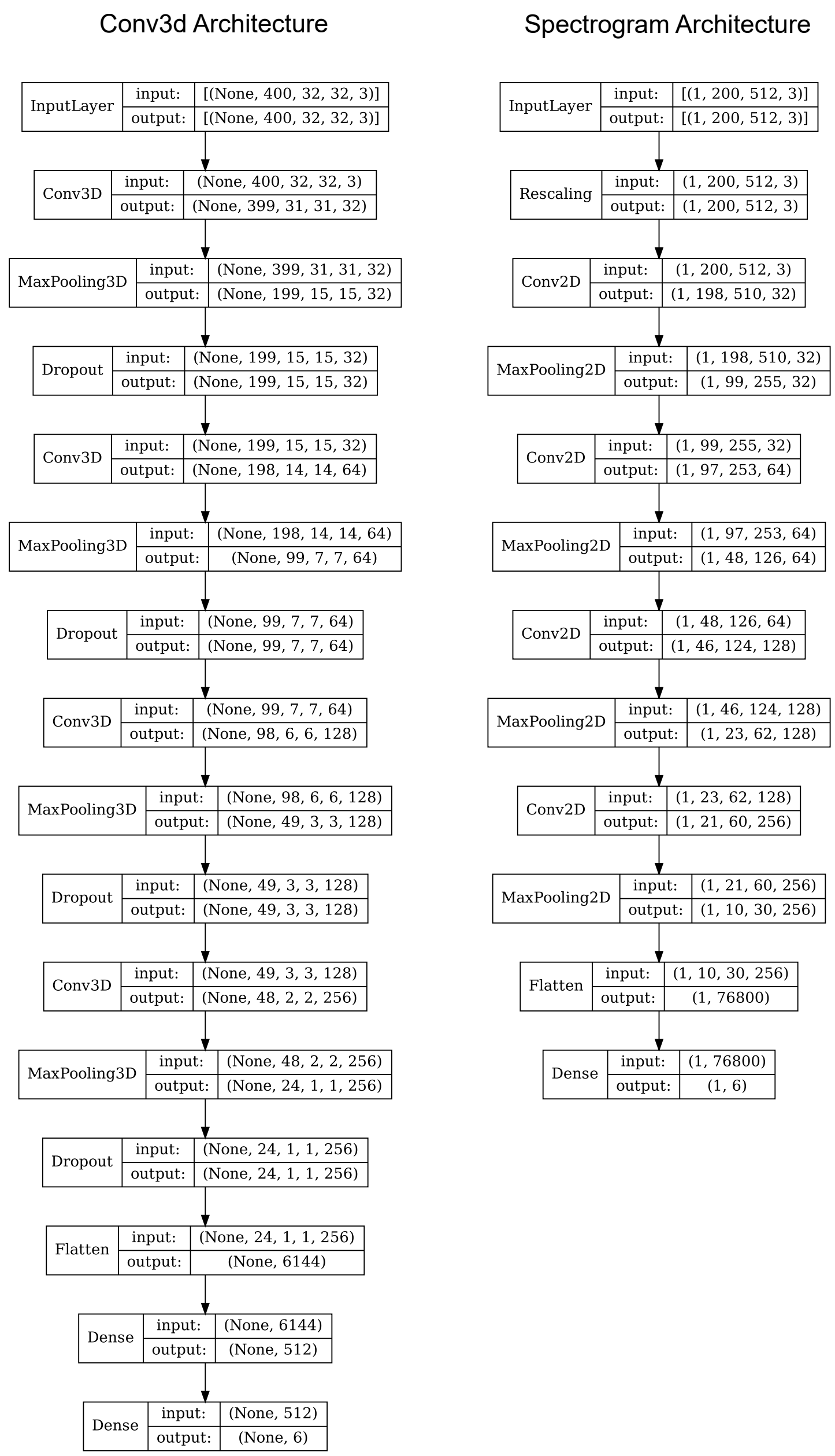

Figure 3: Detailed classifier architectures for Conv3D (left) and Spectrogram (right).

2.1 Training with synthetically generated noise

Conv3D

Homogeneity of Variances Test (Levene's)

F	df1	df2	p
29.1	4	360	< .001

Normality Test (Shapiro-Wilk)

Statistic	p
0.942	< .001

Spectrogram

Homogeneity of Variances Test (Levene's)

F	df1	df2	p
29.1	4	360	< .001

Normality Test (Shapiro-Wilk)

Statistic	p
0.93	< .001

Figure 4: Levene's homogeneity of variance test and Shapiro-Wilk normality test for performance measure of *No-material*, *All-materials*, *Gaussian-noise*, *RandAugment* and *RandAugment with Gaussian-noise* classifiers for Conv3D (left) and Spectrogram (right) architectures. Test results indicate that performance measure is not normally distributed and violates equal variance assumption. We thus analysed the data using non-parametric tests.

2.2 Selecting the best sampling technique

Conv3D

Homogeneity of Variances Test (Levene's)

F	df1	df2	p
0.335	2	4815	0.715

Normality Test (Shapiro-Wilk)

Statistic	p
0.649	< .001

Spectrogram

Homogeneity of Variances Test (Levene's)

F	df1	df2	p
1.48	2	4815	0.22

Normality Test (Shapiro-Wilk)

Statistic	p
0.67	< .001

Figure 5: Levene's homogeneity of variance test and Shapiro-Wilk normality test for performance measure of *Random*, *Quota* and *BO* classifiers. Each distribution is represented by average performance scores of 22 classifiers across 73 materials (1606 data points). Left: results for Conv3D architecture. Right: results for Spectrogram architecture. Test results indicate the data is not normally distributed, and does not violate equal variance assumption. We thus analysed the data using non-parametric tesst.

2.3 Selecting the optimal materials

Conv3D

Bayesian

Homogeneity of Variances Test (Levene's)

F	df1	df2	p
0.335	2	4815	0.715

Normality Test (Shapiro-Wilk)

Statistic	p
0.649	< .001

Quota

Homogeneity of Variances Test (Levene's)

F	df1	df2	p
0.335	2	4815	0.715

Normality Test (Shapiro-Wilk)

Statistic	p
0.649	< .001

Systematic

Homogeneity of Variances Test (Levene's)

F	df1	df2	p
0.914	22	1656	0.57

Normality Test (Shapiro-Wilk)

Statistic	p
0.638	< .001

Spectrogram

Bayesian

Homogeneity of Variances Test (Levene's)

F	df1	df2	p
1.22	22	1656	0.217

Normality Test (Shapiro-Wilk)

Statistic	p
0.623	< .001

Quota

Homogeneity of Variances Test (Levene's)

F	df1	df2	p
1.31	22	1656	0.154

Normality Test (Shapiro-Wilk)

Statistic	p
0.643	< .001

Systematic

Homogeneity of Variances Test (Levene's)

F	df1	df2	p
0.794	22	1656	0.73

Normality Test (Shapiro-Wilk)

Statistic	p
0.671	< .001

Figure 6: Levene's homogeneity of variance test and Shapiro-Wilk normality test for performance measure of *Random*, *Quota* and *BO* sampling for Conv3D (bottom) and Spectrogram (top) architectures tested individually. Test results indicate the data is not normally distributed, and does not violate equal variance assumption. We thus analysed the data using non-parametric tests.

2.4 Random selection

Conv3D #64 materials

Homogeneity of Variances Test (Levene's)

F	df1	df2	p
2.98	2	216	0.053

Normality Test (Shapiro-Wilk)

Statistic	p
0.578	< .001

Spectrogram #44 materials

Homogeneity of Variances Test (Levene's)

F	df1	df2	p
0.193	2	216	0.83

Normality Test (Shapiro-Wilk)

Statistic	p
0.645	< .001

Figure 7: Levene's homogeneity of variance test and Shapiro-Wilk normality test for performance measure of *Random*, *Quota*, and *BO* classifiers trained with 64 materials selected with respective sampling techniques for Conv3D architecture (left) and 44 materials for Spectrogram architecture (right). Test results indicate the data is not normally distributed, and does not violate equal variance assumption. We thus analysed the data using non-parametric tests.

2.5 Quota selection

Conv3D #14 materials

Homogeneity of Variances Test (Levene's)

F	df1	df2	p
0.151	2	216	0.86

Normality Test (Shapiro-Wilk)

Statistic	p
0.59	< .001

Spectrogram #14materials

Homogeneity of Variances Test (Levene's)

F	df1	df2	p
0.13	2	216	0.87

Normality Test (Shapiro-Wilk)

Statistic	p
0.645	< .001

Figure 8: Levene's homogeneity of variance test and Shapiro-Wilk normality test for performance measure of *Random*, *Quota*, and *BO* classifiers trained with 14 materials selected with respective sampling techniques. Left: results for Conv3D architecture. Right: Spectrogram architecture. Test results indicate the data is not normally distributed, and does not violate equal variance assumption, thus we analysed the data using non-parametric statistics.

2.6 BO selection

Conv3D #12 materials

Homogeneity of Variances Test (Levene's)

F	df1	df2	p
0.615	2	216	0.542

Normality Test (Shapiro-Wilk)

Statistic	p
0.633	< .001

Spectrogram #12 materials

Homogeneity of Variances Test (Levene's)

F	df1	df2	p
0.386	2	216	0.68

Normality Test (Shapiro-Wilk)

Statistic	p
0.672	< .001

Figure 9: Levene's homogeneity of variance test and Shapiro-Wilk normality test for performance measure of *Random*, *Quota*, and *BO* classifiers trained with 12 materials selected with respective sampling techniques. Left: results for Conv3D architecture. Right: results for Spectrogram architecture. Test results indicate the data is not normally distributed, and does not violate equal variance assumption. We thus analysed the data using non-parametric tests.

Table 1: Statistical tests for training with synthetically generated noise. Complementing Figure 4 of main paper.

Conv3D

Friedman

χ^2	df	p
153	5	< .001

Post-hoc with Wilcoxon rank test

		W*	p**	Effect Size***
All-materials	No-material	2556	< .001	1.00
	Gaussian-noise	2701	< .001	1.00
	RandAugment	2552	< .001	1.00
	RandAug. with G.-noise	2701	< .001	1.00
	CCycleGAN	2556	< .001	1.00
No-material	Gaussian-noise	980	1	-0.21
	RandAugment	1458	1	0.21
	RandAug. with G.-noise	988	0.705	-0.27
	CCycleGAN	1016	1	-0.23
Gaussian-noise	RandAugment	1504	1	0.25
	RandAug. with G.-noise	1137	1	-0.11
	CCycleGAN	1113	1	-0.18
RandAugment	RandAug. with G.-noise	1087	1	-0.20
	CCycleGAN	1091	1	-0.17
RandAug. with G.-noise	CCycleGAN	1497	1	0.108

Spectrogram

Friedman

χ^2	df	p
155	5	< .001

Post-hoc with Wilcoxon rank test

		W*	p**	Effect Size***
All-materials	No-material	2551	< .001	1.00
	Gaussian-noise	2628	< .001	1.00
	RandAugment	2334	< .001	0.88
	RandAug. with G.-noise	2701	< .001	1.00
	CCycleGAN	2627	< .001	0.95
No-material	Gaussian-noise	320	< .001	-0.73
	RandAugment	529	<.01	-0.55
	RandAug. with G.-noise	924	0.95	-0.26
	CCycleGAN	456	< .001	-0.65
Gaussian-noise	RandAugment	1383	1	0.25
	RandAug. with G.-noise	2154	< .001	0.69
	CCycleGAN	766	0.02	-0.43
RandAugment	RandAug. with G.-noise	1514	1	0.15
	CCycleGAN	916	0.26	-0.322
RandAug. with G.-noise	CCycleGAN	134	< .001	-0.901

* Wilcoxon signed rank test statistic ** Bonferroni corrected p-vaules *** Rank biserial correlation Effect Size

3 Training with synthetically generated noise (RQ1 & RQ2)

In Table 1 you can find statistical tests for training with synthetically generated noise. Tests complement Figure 4 in main paper.

4 Selecting the optimal number of materials (RQ4)

The results for Spectrogram architecture in Figure 11 and Conv3D architecture in Figure 10 compare the optimal classifiers of sampling techniques (determined in Figure 6 main paper) with corresponding classifiers trained with the same number of materials but a different sampling technique. For example, *Bayesian 12-materials* classifier is considered optimal classifier for *Bayesian optimisation sampling* and is compared with *Systematic 12-materials* classifier and *Quota 12-materials* classifier. Similar observations can be made as among both architectures.

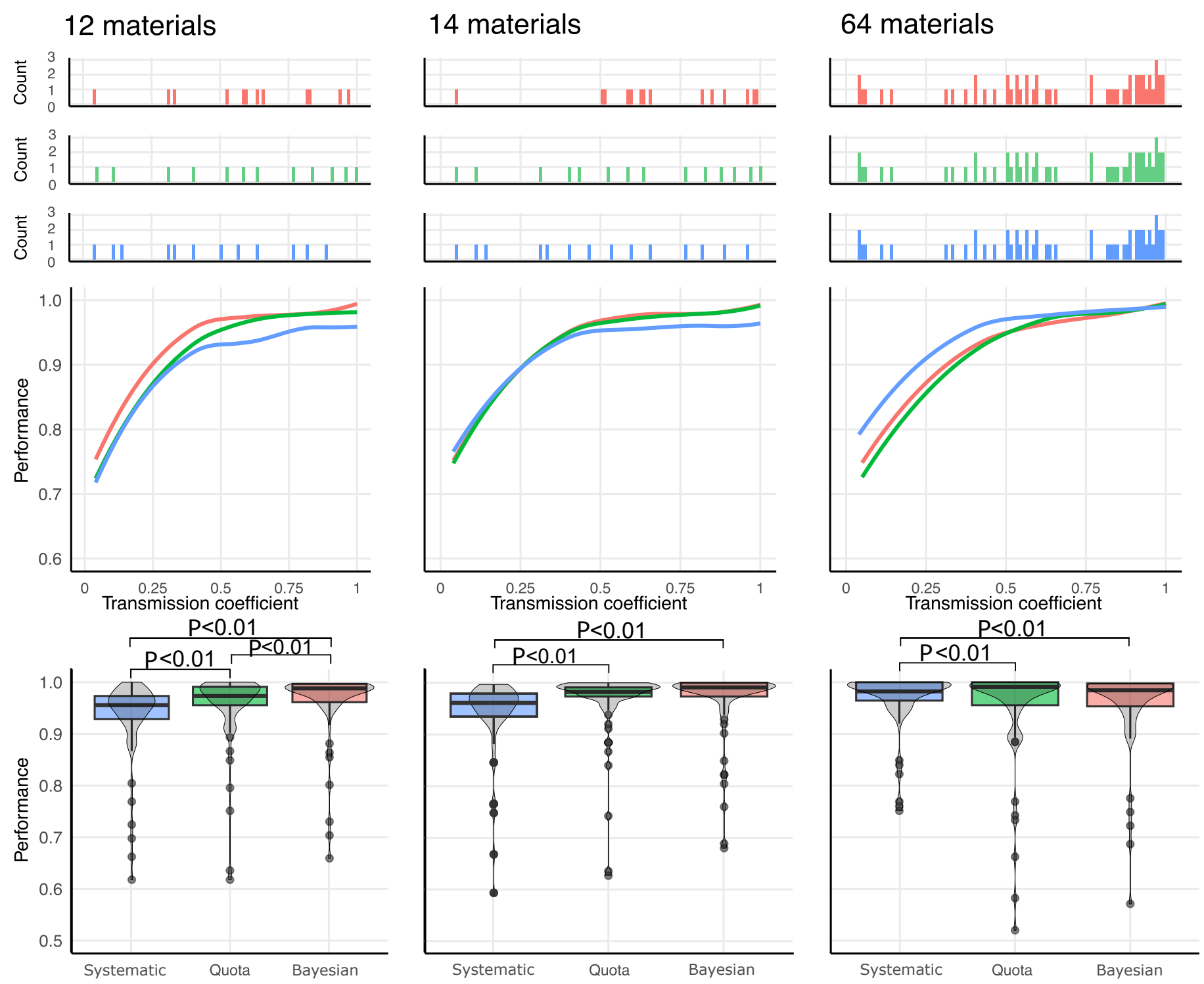

12 materials — Friedman

χ^2	df	p
57.4	2	< .001

14 materials — Friedman

χ^2	df	p
47.6	2	< .001

64 materials — Friedman

χ^2	df	p
13	2	<0.01

Post-hoc with Wilcoxon signed-rank test (12 materials)

		W*	p**	Effect Size***
Bayesian	Quota	1368	<0.01	0.598
	Systematic	1780	<0.01	0.945
Systematic	Quota	469	<0.01	-0.589

Post-hoc with Wilcoxon signed-rank test (14 materials)

		W*	p**	Effect Size***
Quota	Systematic	1654	<0.01	0.749
	Bayesian	464	0.417	-0.242
Systematic	Bayesian	190	<0.01	-0.818

Post-hoc with Wilcoxon signed-rank test (64 materials)

		W*	p**	Effect Size***
Systematic	Quota	950	<0.01	0.551
	Bayesian	981	<0.01	0.538
Quota	Bayesian	366	1	-0.109

* Wilcoxon signed rank test statistic ** Bonferroni corrected p-vaules *** Rank biserial correlation Effect Size

Sampling Techniques: Systematic Quota Bayesian

Figure 10: Comparing the optimal classifier of sampling techniques for Conv3D architecture (i.e., *Bayesian optimisation sampling*—left column, *Quota sampling*—middle column, and *Systematic sampling*—right column) with corresponding classifiers trained with the same number of materials but a different sampling technique. For example, *Bayesian 12-materials* classifier is compared with *Systematic 12-materials* classifier and *Quota 12-materials* classifier. Top histogram plots show what materials were selected in respect to transmission coefficients. Middle plots of trend lines show how the performances of classifiers change with transmission coefficient of materials. The box-plots with violin graph show performance distributions of classifiers. Tables at the bottom show Friedman and Wilcoxon signed-rank test results comparing classifier performance.

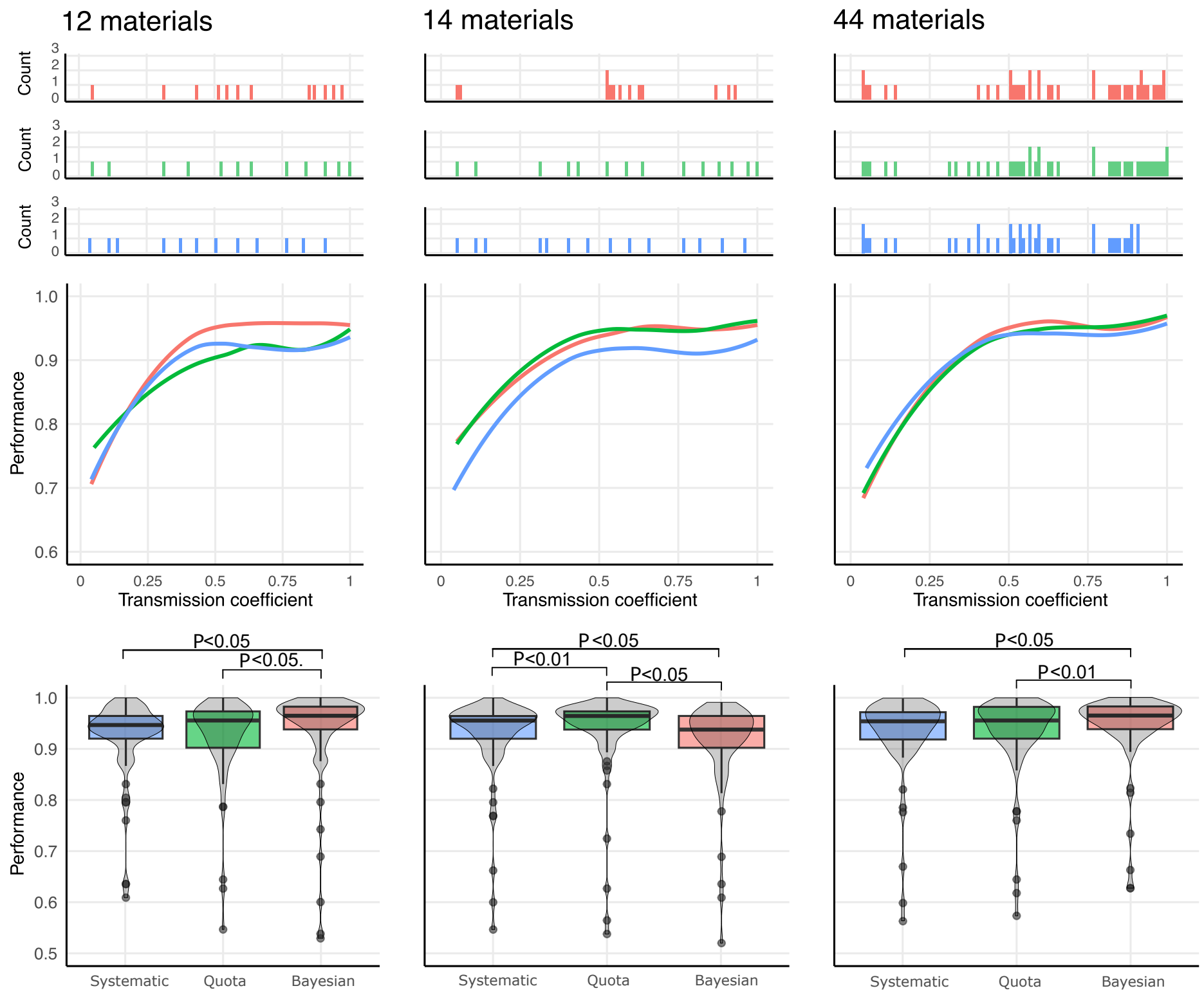

Friedman (12 materials)

χ^2	df	p
10.4	2	<0.01

Post-hoc with Wilcoxon signed-rank test (12 materials)

		W*	p**	Effect Size***
Bayesian	Quota	1551	<0.05	-0.102
	Systematic	1581	<0.05	-0.207
Systematic	Quota	1131	1	-0.13

Friedman (14 materials)

χ^2	df	p
23.8	2	< .001

Post-hoc with Wilcoxon signed-rank test (14 materials)

		W*	p**	Effect Size***
Quota	Systematic	1470	<0.05	0.37
	Bayesian	1716	<0.01	0.702
Systematic	Bayesian	1474	<0.05	0.417

Friedman (44 materials)

χ^2	df	p
6.95	2	<0.05

Post-hoc with Wilcoxon signed-rank test (44 materials)

		W*	p**	Effect Size***
Systematic	Quota	1163	1	-0.009
	Bayesian	593	<0.05	-0.412
Quota	Bayesian	540	<0.01	-0.447

* Wilcoxon signed rank test statistic ** Bonferroni corrected p-vaules *** Rank biserial correlation Effect Size

Sampling Techniques: Systematic Quota Bayesian

Figure 11: Comparing the optimal classifier of sampling techniques for Spectrogram architecture (i.e., *Random*—left column, *Quota*—middle column, and *BO*—right column) with corresponding classifiers trained with the same number of materials but a different sampling technique. Top histogram plots show what materials were selected in respect to transmission coefficients. Middle trend plots show how the performances of classifiers change with transmission coefficient of materials. The bottom box-plots show performance distributions of classifiers. Tables at the bottom show Friedman and Wilcoxon signed-rank test results comparing classifier performance.

5 Chosen materials

In Table 7 below you can find what materials were chosen by different sampling techniques.

6 Gesture classification performance

In Table 2, Table 3, Table 4, Table 5 and Table 6 you can find standard metrics, such as ACC, AUC, Micro-F1 and Performance score for various models we trained and evaluated.

Table 7: Chosen materials. Complementary to Figure 7 of main paper.

			Conv3D architecture									Spectrogram architecture								
Material ID	T_c	Thickness (mm)	Bayesian 12	Quota 12	Systematic 12	Bayesian 14	Quota 14	Systematic 14	Bayesian 64	Quota 64	Systematic 64	Bayesian 12	Quota 12	Systematic 12	Bayesian 14	Quota 14	Systematic 14	Bayesian 44	Quota 44	Systematic 44
2	1.00	4		✓			✓		✓	✓			✓		✓	✓		✓	✓	
3	1.00	3							✓	✓								✓	✓	
4	1.00	20							✓	✓								✓		
6	1.00	10							✓	✓										
7	1.00	50							✓	✓	✓									
9	1.00	50									✓									
28	1.00	2																		
29	1.00	5									✓									
32	1.00	5									✓									
34	1.00	0.1																		
38	1.00	-									✓									
67	1.00	20																		
68	1.00	30																		
70	1.00	30																		
35	0.99	1.1				✓			✓	✓	✓							✓	✓	
63	0.99	20							✓	✓	✓							✓		
17	0.98	10				✓			✓	✓	✓							✓	✓	
69	0.98	20							✓	✓	✓									
14	0.97	40	✓				✓		✓	✓	✓	✓				✓		✓	✓	
39	0.97	-							✓	✓	✓									
42	0.97	20							✓	✓	✓									
41	0.96	-		✓		✓		✓	✓	✓	✓		✓				✓	✓	✓	
16	0.95	0.5							✓	✓	✓								✓	
27	0.95	0.8							✓	✓	✓									
15	0.94	0.5	✓						✓	✓	✓	✓						✓	✓	
13	0.93	0.2							✓	✓	✓				✓			✓	✓	
66	0.93	0.5							✓	✓	✓									
20	0.92	0.2					✓		✓	✓	✓					✓		✓	✓	
75	0.92	0.5							✓	✓	✓							✓		
30	0.91	0.3		✓					✓	✓	✓	✓	✓	✓	✓			✓	✓	✓
40	0.91	-							✓	✓	✓									✓
24	0.89	0.5				✓			✓	✓	✓							✓	✓	✓
33	0.89	0.5			✓			✓	✓	✓	✓						✓			✓
8	0.88	0.5					✓		✓	✓	✓					✓		✓	✓	✓
76	0.87	0.5							✓	✓	✓	✓			✓			✓	✓	✓
12	0.85	0.5				✓			✓	✓	✓	✓						✓	✓	✓
31	0.84	0.5		✓					✓	✓	✓		✓					✓	✓	✓
64	0.83	0.4	✓				✓		✓	✓	✓			✓		✓		✓	✓	✓
19	0.82	0.4	✓		✓	✓		✓	✓	✓	✓						✓	✓	✓	✓
36	0.77	0.5		✓	✓		✓		✓	✓	✓		✓	✓		✓		✓	✓	✓
77	0.77	0.5						✓	✓	✓	✓						✓	✓	✓	✓
57	0.66	0.5	✓			✓		✓	✓	✓	✓			✓			✓	✓	✓	✓

Continued on next page

			Conv3D architecture (cont.)									Spectrogram architecture (cont.)								
Material ID	T_c	Thickness (mm)	Bayesian 12	Quota 12	Systematic 12	Bayesian 14	Quota 14	Systematic 14	Bayesian 64	Quota 64	Systematic 64	Bayesian 12	Quota 12	Systematic 12	Bayesian 14	Quota 14	Systematic 14	Bayesian 44	Quota 44	Systematic 44
44	0.64	0.5	✓	✓	✓	✓	✓		✓	✓	✓	✓	✓		✓	✓		✓	✓	✓
37	0.63	0.5				✓			✓	✓	✓				✓			✓	✓	✓
46	0.60	0.5	✓			✓		✓	✓	✓	✓				✓			✓	✓	✓
59	0.60	0.5							✓	✓	✓						✓	✓	✓	✓
47	0.59	0.5	✓	✓		✓	✓		✓	✓	✓	✓	✓	✓		✓			✓	✓
43	0.57	0.5			✓				✓	✓	✓				✓			✓	✓	✓
54	0.57	0.5							✓	✓	✓							✓	✓	✓
60	0.55	0.5							✓	✓	✓	✓			✓			✓	✓	✓
26	0.54	0.5							✓	✓	✓				✓		✓	✓	✓	✓
49	0.54	0.5						✓	✓	✓	✓									✓
1	0.53	0.5	✓	✓			✓		✓	✓	✓		✓		✓	✓		✓	✓	✓
50	0.53	0.5							✓	✓	✓				✓					✓
52	0.53	0.5							✓	✓	✓									✓
53	0.53	0.5							✓	✓	✓									✓
58	0.53	0.5							✓	✓	✓									✓
55	0.52	0.5				✓			✓	✓	✓	✓						✓	✓	✓
45	0.51	0.5				✓			✓	✓	✓							✓	✓	✓
62	0.51	0.5			✓				✓	✓	✓			✓				✓		✓
48	0.46	0.5						✓	✓	✓	✓						✓	✓	✓	✓
79	0.43	0.5					✓		✓	✓	✓	✓		✓		✓		✓	✓	✓
51	0.40	0.5		✓	✓		✓		✓	✓	✓		✓			✓		✓	✓	✓
56	0.40	0.5						✓	✓	✓	✓						✓			✓
10	0.37	0.5							✓	✓	✓			✓					✓	✓
61	0.33	0.5	✓		✓			✓	✓	✓	✓						✓		✓	✓
18	0.31	0.5	✓	✓	✓		✓	✓	✓	✓	✓	✓	✓	✓		✓	✓		✓	✓
5	0.14	0.5																		
21	0.14	0.5			✓			✓	✓	✓	✓			✓			✓	✓	✓	✓
80	0.11	0.5		✓	✓		✓	✓	✓	✓	✓		✓	✓		✓	✓	✓	✓	✓
72	0.06	0.5							✓	✓	✓				✓			✓	✓	✓
78	0.05	0.5		✓		✓	✓	✓	✓	✓	✓	✓	✓		✓	✓	✓	✓	✓	✓
73	0.04	0.5	✓						✓	✓	✓							✓	✓	✓
82	0.04	0.5			✓				✓	✓	✓			✓				✓		✓

References

[1] K. Čopič Pucihar, N. T. Attygalle, M. Kljun, C. Sandor, and L. A. Leiva, "Solids on soli: Millimetre-wave radar sensing through materials," *Proc. ACM Hum. Comput. Interact.*, vol. 6, no. EICS, pp. 1–19, 2022.

[2] N. T. Attygalle, L. A. Leiva, M. Kljun, C. Sandor, A. Plopski, H. Kato, and K. Čopič Pucihar, "No interface, no problem: gesture recognition on physical objects using radar sensing," *Sensors*, vol. 21, no. 17, 2021.

Table 2: Gesture classification performance of the Conv3D models trained on reduced set of matierls and evaluated on materials 1–82, reported using Accuracy (ACC), Area Under the ROC Curve (AUC), Micro-F1 score (F1), and the combined performance metric $Perf = (ACC + AUC)/2$. The table continues on the next page.

Material ID	Conv3D Bayesian 12				Conv3D Quota 14				Conv3D Systematic 64			
	ACC	AUC	F1	Perf.	ACC	AUC	F1	Perf.	ACC	AUC	F1	Perf.
1	85.6	91.3	85.6	88.4	85.6	91.3	85.6	88.4	76.7	86.0	76.7	81.3
2	98.9	99.3	98.9	99.1	98.9	99.3	98.9	99.1	90.0	94.0	90.0	92.0
3	100.0	100.0	100.0	100.0	100.0	100.0	100.0	100.0	100.0	100.0	100.0	100.0
4	97.8	98.7	97.8	98.2	97.8	98.7	97.8	98.2	97.8	98.7	97.8	98.2
6	100.0	100.0	100.0	100.0	100.0	100.0	100.0	100.0	98.9	99.3	98.9	99.1
7	100.0	100.0	100.0	100.0	95.6	97.3	95.6	96.4	96.7	98.0	96.7	97.3
8	96.7	98.0	96.7	97.3	96.7	98.0	96.7	97.3	87.8	92.7	87.8	90.2
9	100.0	100.0	100.0	100.0	100.0	100.0	100.0	100.0	100.0	100.0	100.0	100.0
10	95.6	97.3	95.6	96.4	94.4	96.7	94.4	95.6	87.8	92.7	87.8	90.2
12	100.0	100.0	100.0	100.0	98.9	99.3	98.9	99.1	98.9	99.3	98.9	99.1
13	100.0	100.0	100.0	100.0	100.0	100.0	100.0	100.0	100.0	100.0	100.0	100.0
14	97.8	98.7	97.8	98.2	98.9	99.3	98.9	99.1	93.3	96.0	93.3	94.7
15	98.9	99.3	98.9	99.1	98.9	99.3	98.9	99.1	95.6	97.3	95.6	96.4
16	98.9	99.3	98.9	99.1	97.8	98.7	97.8	98.2	98.9	99.3	98.9	99.1
17	98.9	99.3	98.9	99.1	98.9	99.3	98.9	99.1	100.0	100.0	100.0	100.0
18	94.4	96.7	94.4	95.6	93.3	96.0	93.3	94.7	90.0	94.0	90.0	92.0
19	97.8	98.7	97.8	98.2	94.4	96.7	94.4	95.6	92.2	95.3	92.2	93.8
20	95.6	97.3	95.6	96.4	88.9	93.3	88.9	91.1	90.0	94.0	90.0	92.0
21	94.4	96.7	94.4	95.6	92.2	95.3	92.2	93.8	90.0	94.0	90.0	92.0
24	100.0	100.0	100.0	100.0	96.7	98.0	96.7	97.3	97.8	98.7	97.8	98.2
26	83.3	90.0	83.3	86.7	83.3	90.0	83.3	86.7	88.9	93.3	88.9	91.1
27	98.9	99.3	98.9	99.1	96.7	98.0	96.7	97.3	92.2	95.3	92.2	93.8
28	100.0	100.0	100.0	100.0	100.0	100.0	100.0	100.0	98.9	99.3	98.9	99.1
29	97.8	98.7	97.8	98.2	98.9	99.3	98.9	99.1	92.2	95.3	92.2	93.8
30	100.0	100.0	100.0	100.0	98.9	99.3	98.9	99.1	98.9	99.3	98.9	99.1
31	93.3	96.0	93.3	94.7	100.0	100.0	100.0	100.0	98.9	99.3	98.9	99.1
32	100.0	100.0	100.0	100.0	100.0	100.0	100.0	100.0	98.9	99.3	98.9	99.1
33	95.6	97.3	95.6	96.4	98.9	99.3	98.9	99.1	94.4	96.7	94.4	95.6
34	97.8	98.7	97.8	98.2	98.9	99.3	98.9	99.1	100.0	100.0	100.0	100.0
35	98.9	99.3	98.9	99.1	98.9	99.3	98.9	99.1	100.0	100.0	100.0	100.0
36	98.9	99.3	98.9	99.1	100.0	100.0	100.0	100.0	100.0	100.0	100.0	100.0
37	98.9	99.3	98.9	99.1	100.0	100.0	100.0	100.0	97.8	98.7	97.8	98.2
38	100.0	100.0	100.0	100.0	100.0	100.0	100.0	100.0	97.8	98.7	97.8	98.2
39	98.9	99.3	98.9	99.1	98.9	99.3	98.9	99.1	94.4	96.7	94.4	95.6
40	100.0	100.0	100.0	100.0	98.9	99.3	98.9	99.1	97.8	98.7	97.8	98.2
41	100.0	100.0	100.0	100.0	98.9	99.3	98.9	99.1	97.8	98.7	97.8	98.2
42	98.9	99.3	98.9	99.1	97.8	98.7	97.8	98.2	97.8	98.7	97.8	98.2

Table 2: Continued from previous page.

Material ID	Conv3D Bayesian 12				Conv3D Quota 14				Conv3D Systematic 64			
	ACC	AUC	F1	Perf.	ACC	AUC	F1	Perf.	ACC	AUC	F1	Perf.
43	96.7	98.0	96.7	97.3	96.7	98.0	96.7	97.3	91.1	94.7	91.1	92.9
44	98.9	99.3	98.9	99.1	100.0	100.0	100.0	100.0	91.1	94.7	91.1	92.9
45	97.8	98.7	97.8	98.2	96.7	98.0	96.7	97.3	92.2	95.3	92.2	93.8
46	98.9	99.3	98.9	99.1	97.8	98.7	97.8	98.2	93.3	96.0	93.3	94.7
47	98.9	99.3	98.9	99.1	100.0	100.0	100.0	100.0	97.8	98.7	97.8	98.2
48	96.7	98.0	96.7	97.3	97.8	98.7	97.8	98.2	94.4	96.7	94.4	95.6
49	100.0	100.0	100.0	100.0	98.9	99.3	98.9	99.1	94.4	96.7	94.4	95.6
50	97.8	98.7	97.8	98.2	98.9	99.3	98.9	99.1	83.3	90.0	83.3	86.7
51	95.6	97.3	95.6	96.4	97.8	98.7	97.8	98.2	87.8	92.7	87.8	90.2
52	97.8	98.7	97.8	98.2	98.9	99.3	98.9	99.1	95.6	97.3	95.6	96.4
53	97.8	98.7	97.8	98.2	97.8	98.7	97.8	98.2	92.2	95.3	92.2	93.8
54	100.0	100.0	100.0	100.0	96.7	98.0	96.7	97.3	95.6	97.3	95.6	96.4
55	98.9	99.3	98.9	99.1	96.7	98.0	96.7	97.3	92.2	95.3	92.2	93.8
56	100.0	100.0	100.0	100.0	93.3	96.0	93.3	94.7	93.3	96.0	93.3	94.7
57	98.9	99.3	98.9	99.1	95.6	97.3	95.6	96.4	88.9	93.3	88.9	91.1
58	90.0	94.0	90.0	92.0	85.6	91.3	85.6	88.4	80.0	88.0	80.0	84.0
59	97.8	98.7	97.8	98.2	97.8	98.7	97.8	98.2	93.3	96.0	93.3	94.7
60	98.9	99.3	98.9	99.1	96.7	98.0	96.7	97.3	94.4	96.7	94.4	95.6
61	97.8	98.7	97.8	98.2	94.4	96.7	94.4	95.6	94.4	96.7	94.4	95.6
62	100.0	100.0	100.0	100.0	97.8	98.7	97.8	98.2	97.8	98.7	97.8	98.2
63	100.0	100.0	100.0	100.0	98.9	99.3	98.9	99.1	97.8	98.7	97.8	98.2
64	100.0	100.0	100.0	100.0	98.9	99.3	98.9	99.1	97.8	98.7	97.8	98.2
66	98.9	99.3	98.9	99.1	100.0	100.0	100.0	100.0	100.0	100.0	100.0	100.0
67	98.9	99.3	98.9	99.1	100.0	100.0	100.0	100.0	100.0	100.0	100.0	100.0
68	100.0	100.0	100.0	100.0	98.9	99.3	98.9	99.1	96.7	98.0	96.7	97.3
69	95.6	97.3	95.6	96.4	100.0	100.0	100.0	100.0	100.0	100.0	100.0	100.0
70	98.9	99.3	98.9	99.1	96.7	98.0	96.7	97.3	95.6	97.3	95.6	96.4
72	66.7	80.0	66.7	73.3	67.8	80.7	67.8	74.2	61.1	76.7	61.1	68.9
73	57.8	74.7	57.8	66.2	54.4	72.7	54.4	63.6	62.2	77.3	62.2	69.8
75	100.0	100.0	100.0	100.0	97.8	98.7	97.8	98.2	98.9	99.3	98.9	99.1
76	95.6	97.3	95.6	96.4	97.8	98.7	97.8	98.2	97.8	98.7	97.8	98.2
77	93.3	96.0	93.3	94.7	95.6	97.3	95.6	96.4	88.9	93.3	88.9	91.1
78	75.6	85.3	75.6	80.4	80.0	88.0	80.0	84.0	75.6	85.3	75.6	80.4
79	93.3	96.0	93.3	94.7	96.7	98.0	96.7	97.3	86.7	92.0	86.7	89.3
80	82.2	89.3	82.2	85.8	90.0	94.0	90.0	92.0	74.4	84.7	74.4	79.6
82	63.3	78.0	63.3	70.7	53.3	72.0	53.3	62.7	55.6	73.3	55.6	64.4
	**95.8**	**97.5**	**95.8**	**96.6**	**95.3**	**97.2**	**95.3**	**96.3**	**92.6**	**95.6**	**92.6**	**94.1**

Table 3: Gesture classification performance of the Spectrogram models trained on reduced set of matierls and evaluated on materials 1–82, reported using Accuracy (ACC), Area Under the ROC Curve (AUC), Micro-F1 score (F1), and the combined performance metric $Perf = (ACC + AUC)/2$. The table continues on the next page.

Material ID	Spectrogram Bayesian 12				Spectrogram Quota 14				Spectrogram Systematic 44			
	ACC	AUC	F1	Perf.	ACC	AUC	F1	Perf.	ACC	AUC	F1	Perf.
1	75.6	85.3	75.6	80.4	86.7	92.0	86.7	89.3	72.2	83.3	72.2	77.8
2	87.8	92.7	87.8	90.2	95.6	97.3	95.6	96.4	74.4	84.7	74.4	79.6
3	96.7	98.0	96.7	97.3	96.7	98.0	96.7	97.3	91.1	94.7	91.1	92.9
4	97.8	98.7	97.8	98.2	97.8	98.7	97.8	98.2	93.3	96.0	93.3	94.7
6	96.7	98.0	96.7	97.3	94.4	96.7	94.4	95.6	92.2	95.3	92.2	93.8
7	96.7	98.0	96.7	97.3	97.8	98.7	97.8	98.2	93.3	96.0	93.3	94.7
8	81.1	88.7	81.1	84.9	95.6	97.3	95.6	96.4	80.0	88.0	80.0	84.0
9	97.8	98.7	97.8	98.2	95.6	97.3	95.6	96.4	95.6	97.3	95.6	96.4
10	86.7	92.0	86.7	89.3	86.7	92.0	86.7	89.3	81.1	88.7	81.1	84.9
12	100.0	100.0	100.0	100.0	97.8	98.7	97.8	98.2	91.1	94.7	91.1	92.9
13	95.6	97.3	95.6	96.4	98.9	99.3	98.9	99.1	95.6	97.3	95.6	96.4
14	94.4	96.7	94.4	95.6	88.9	93.3	88.9	91.1	76.7	86.0	76.7	81.3
15	94.4	96.7	94.4	95.6	85.6	91.3	85.6	88.4	80.0	88.0	80.0	84.0
16	94.4	96.7	94.4	95.6	93.3	96.0	93.3	94.7	84.4	90.7	84.4	87.6
17	96.7	98.0	96.7	97.3	97.8	98.7	97.8	98.2	88.9	93.3	88.9	91.1
18	88.9	93.3	88.9	91.1	90.0	94.0	90.0	92.0	83.3	90.0	83.3	86.7
19	94.4	96.7	94.4	95.6	87.8	92.7	87.8	90.2	82.2	89.3	82.2	85.8
20	93.3	96.0	93.3	94.7	92.2	95.3	92.2	93.8	88.9	93.3	88.9	91.1
21	87.8	92.7	87.8	90.2	83.3	90.0	83.3	86.7	87.8	92.7	87.8	90.2
24	97.8	98.7	97.8	98.2	92.2	95.3	92.2	93.8	95.6	97.3	95.6	96.4
26	84.4	90.7	84.4	87.6	84.4	90.7	84.4	87.6	80.0	88.0	80.0	84.0
27	94.4	96.7	94.4	95.6	95.6	97.3	95.6	96.4	85.6	91.3	85.6	88.4
28	95.6	97.3	95.6	96.4	96.7	98.0	96.7	97.3	84.4	90.7	84.4	87.6
29	90.0	94.0	90.0	92.0	93.3	96.0	93.3	94.7	90.0	94.0	90.0	92.0
30	98.9	99.3	98.9	99.1	97.8	98.7	97.8	98.2	95.6	97.3	95.6	96.4
31	97.8	98.7	97.8	98.2	88.9	93.3	88.9	91.1	94.4	96.7	94.4	95.6
32	93.3	96.0	93.3	94.7	96.7	98.0	96.7	97.3	87.8	92.7	87.8	90.2
33	95.6	97.3	95.6	96.4	96.7	98.0	96.7	97.3	91.1	94.7	91.1	92.9
34	93.3	96.0	93.3	94.7	96.7	98.0	96.7	97.3	92.2	95.3	92.2	93.8
35	93.3	96.0	93.3	94.7	97.8	98.7	97.8	98.2	82.2	89.3	82.2	85.8
36	96.7	98.0	96.7	97.3	97.8	98.7	97.8	98.2	96.7	98.0	96.7	97.3
37	92.2	95.3	92.2	93.8	94.4	96.7	94.4	95.6	94.4	96.7	94.4	95.6
38	97.8	98.7	97.8	98.2	100.0	100.0	100.0	100.0	90.0	94.0	90.0	92.0
39	96.7	98.0	96.7	97.3	97.8	98.7	97.8	98.2	93.3	96.0	93.3	94.7
40	93.3	96.0	93.3	94.7	97.8	98.7	97.8	98.2	91.1	94.7	91.1	92.9
41	91.1	94.7	91.1	92.9	94.4	96.7	94.4	95.6	78.9	87.3	78.9	83.1
42	93.3	96.0	93.3	94.7	90.0	94.0	90.0	92.0	90.0	94.0	90.0	92.0

Table 3: Continued from previous page.

Material ID	Conv3D Bayesian 12				Conv3D Quota 14				Conv3D Systematic 64			
	ACC	AUC	F1	Perf.	ACC	AUC	F1	Perf.	ACC	AUC	F1	Perf.
43	96.7	98.0	96.7	97.3	96.7	98.0	96.7	97.3	91.1	94.7	91.1	92.9
44	98.9	99.3	98.9	99.1	100.0	100.0	100.0	100.0	91.1	94.7	91.1	92.9
45	97.8	98.7	97.8	98.2	96.7	98.0	96.7	97.3	92.2	95.3	92.2	93.8
46	98.9	99.3	98.9	99.1	97.8	98.7	97.8	98.2	93.3	96.0	93.3	94.7
47	98.9	99.3	98.9	99.1	100.0	100.0	100.0	100.0	97.8	98.7	97.8	98.2
48	96.7	98.0	96.7	97.3	97.8	98.7	97.8	98.2	94.4	96.7	94.4	95.6
49	100.0	100.0	100.0	100.0	98.9	99.3	98.9	99.1	94.4	96.7	94.4	95.6
50	97.8	98.7	97.8	98.2	98.9	99.3	98.9	99.1	83.3	90.0	83.3	86.7
51	95.6	97.3	95.6	96.4	97.8	98.7	97.8	98.2	87.8	92.7	87.8	90.2
52	97.8	98.7	97.8	98.2	98.9	99.3	98.9	99.1	95.6	97.3	95.6	96.4
53	97.8	98.7	97.8	98.2	97.8	98.7	97.8	98.2	92.2	95.3	92.2	93.8
54	100.0	100.0	100.0	100.0	96.7	98.0	96.7	97.3	95.6	97.3	95.6	96.4
55	98.9	99.3	98.9	99.1	96.7	98.0	96.7	97.3	92.2	95.3	92.2	93.8
56	100.0	100.0	100.0	100.0	93.3	96.0	93.3	94.7	93.3	96.0	93.3	94.7
57	98.9	99.3	98.9	99.1	95.6	97.3	95.6	96.4	88.9	93.3	88.9	91.1
58	90.0	94.0	90.0	92.0	85.6	91.3	85.6	88.4	80.0	88.0	80.0	84.0
59	97.8	98.7	97.8	98.2	97.8	98.7	97.8	98.2	93.3	96.0	93.3	94.7
60	98.9	99.3	98.9	99.1	96.7	98.0	96.7	97.3	94.4	96.7	94.4	95.6
61	97.8	98.7	97.8	98.2	94.4	96.7	94.4	95.6	94.4	96.7	94.4	95.6
62	100.0	100.0	100.0	100.0	97.8	98.7	97.8	98.2	97.8	98.7	97.8	98.2
63	100.0	100.0	100.0	100.0	98.9	99.3	98.9	99.1	97.8	98.7	97.8	98.2
64	100.0	100.0	100.0	100.0	98.9	99.3	98.9	99.1	97.8	98.7	97.8	98.2
66	98.9	99.3	98.9	99.1	100.0	100.0	100.0	100.0	100.0	100.0	100.0	100.0
67	98.9	99.3	98.9	99.1	100.0	100.0	100.0	100.0	100.0	100.0	100.0	100.0
68	100.0	100.0	100.0	100.0	98.9	99.3	98.9	99.1	96.7	98.0	96.7	97.3
69	95.6	97.3	95.6	96.4	100.0	100.0	100.0	100.0	100.0	100.0	100.0	100.0
70	98.9	99.3	98.9	99.1	96.7	98.0	96.7	97.3	95.6	97.3	95.6	96.4
72	66.7	80.0	66.7	73.3	67.8	80.7	67.8	74.2	61.1	76.7	61.1	68.9
73	57.8	74.7	57.8	66.2	54.4	72.7	54.4	63.6	62.2	77.3	62.2	69.8
75	100.0	100.0	100.0	100.0	97.8	98.7	97.8	98.2	98.9	99.3	98.9	99.1
76	95.6	97.3	95.6	96.4	97.8	98.7	97.8	98.2	97.8	98.7	97.8	98.2
77	93.3	96.0	93.3	94.7	95.6	97.3	95.6	96.4	88.9	93.3	88.9	91.1
78	75.6	85.3	75.6	80.4	80.0	88.0	80.0	84.0	75.6	85.3	75.6	80.4
79	93.3	96.0	93.3	94.7	96.7	98.0	96.7	97.3	86.7	92.0	86.7	89.3
80	82.2	89.3	82.2	85.8	90.0	94.0	90.0	92.0	74.4	84.7	74.4	79.6
82	63.3	78.0	63.3	70.7	53.3	72.0	53.3	62.7	55.6	73.3	55.6	64.4
	**95.8**	**97.5**	**95.8**	**96.6**	**95.3**	**97.2**	**95.3**	**96.3**	**92.6**	**95.6**	**92.6**	**94.1**

Table 4: Gesture classification performance of the Conv3D and Spectrogram models trained on no-matierls or all-materials and evaluated on materials 1–82, reported using Accuracy (ACC), Area Under the ROC Curve (AUC), Micro-F1 score (F1), and the combined performance metric $Perf = (ACC + AUC)/2$. The table continues on the next page.

Material ID	Conv3D No Material				Conv3D All Materials				Spectrogram No Material				Spectrogram All Materials			
	ACC	AUC	F1	Perf.	ACC	AUC	F1	Perf.	ACC	AUC	F1	Perf.	ACC	AUC	F1	Perf.
1	42.2	65.3	38.2	53.8	78.9	87.3	67.3	83.1	47.8	68.7	42.2	58.2	73.3	84.0	62.3	78.7
2	81.1	88.7	69.4	84.9	100.0	100.0	100.0	100.0	76.7	86.0	65.2	81.3	91.1	94.7	80.4	92.9
3	94.4	96.7	85.0	95.6	100.0	100.0	100.0	100.0	92.2	95.3	81.8	93.8	98.9	99.3	93.9	99.1
4	94.4	96.7	85.0	95.6	98.9	99.3	93.9	99.1	84.4	90.7	72.8	87.6	100.0	100.0	100.0	100.0
6	86.7	92.0	75.1	89.3	98.9	99.3	93.9	99.1	81.1	88.7	69.4	84.9	97.8	98.7	91.0	98.2
7	92.2	95.3	81.8	93.8	100.0	100.0	100.0	100.0	91.1	94.7	80.4	92.9	98.9	99.3	93.9	99.1
8	83.3	90.0	71.6	86.7	97.8	98.7	91.0	98.2	65.6	79.3	55.8	72.4	88.9	93.3	77.7	91.1
9	88.9	93.3	77.7	91.1	100.0	100.0	100.0	100.0	88.9	93.3	77.7	91.1	98.9	99.3	93.9	99.1
10	35.6	61.3	33.6	48.4	96.7	98.0	88.8	97.3	37.8	62.7	35.1	50.2	87.8	92.7	76.4	90.2
12	82.2	89.3	70.5	85.8	100.0	100.0	100.0	100.0	80.0	88.0	68.3	84.0	95.6	97.3	86.8	96.4
13	100.0	100.0	100.0	100.0	100.0	100.0	100.0	100.0	95.6	97.3	86.8	96.4	100.0	100.0	100.0	100.0
14	90.0	94.0	79.0	92.0	98.9	99.3	93.9	99.1	72.2	83.3	61.3	77.8	93.3	96.0	83.4	94.7
15	93.3	96.0	83.4	94.7	100.0	100.0	100.0	100.0	76.7	86.0	65.2	81.3	91.1	94.7	80.4	92.9
16	91.1	94.7	80.4	92.9	100.0	100.0	100.0	100.0	78.9	87.3	67.3	83.1	94.4	96.7	85.0	95.6
17	97.8	98.7	91.0	98.2	100.0	100.0	100.0	100.0	91.1	94.7	80.4	92.9	96.7	98.0	88.8	97.3
18	34.4	60.7	32.8	47.6	96.7	98.0	88.8	97.3	40.0	64.0	36.7	52.0	86.7	92.0	75.1	89.3
19	83.3	90.0	71.6	86.7	97.8	98.7	91.0	98.2	67.8	80.7	57.6	74.2	88.9	93.3	77.7	91.1
20	86.7	92.0	75.1	89.3	100.0	100.0	100.0	100.0	62.2	77.3	53.1	69.8	93.3	96.0	83.4	94.7
21	32.2	59.3	31.3	45.8	92.2	95.3	81.8	93.8	44.4	66.7	39.8	55.6	83.3	90.0	71.6	86.7
24	88.9	93.3	77.7	91.1	100.0	100.0	100.0	100.0	93.3	96.0	83.4	94.7	96.7	98.0	88.8	97.3
26	54.4	72.7	47.1	63.6	96.7	98.0	88.8	97.3	56.7	74.0	48.8	65.3	90.0	94.0	79.0	92.0
27	92.2	95.3	81.8	93.8	100.0	100.0	100.0	100.0	80.0	88.0	68.3	84.0	94.4	96.7	85.0	95.6
28	96.7	98.0	88.8	97.3	100.0	100.0	100.0	100.0	96.7	98.0	88.8	97.3	96.7	98.0	88.8	97.3
29	92.2	95.3	81.8	93.8	100.0	100.0	100.0	100.0	82.2	89.3	70.5	85.8	96.7	98.0	88.8	97.3
30	84.4	90.7	72.8	87.6	100.0	100.0	100.0	100.0	88.9	93.3	77.7	91.1	94.4	96.7	85.0	95.6
31	88.9	93.3	77.7	91.1	98.9	99.3	93.9	99.1	91.1	94.7	80.4	92.9	94.4	96.7	85.0	95.6
32	90.0	94.0	79.0	92.0	100.0	100.0	100.0	100.0	84.4	90.7	72.8	87.6	97.8	98.7	91.0	98.2
33	87.8	92.7	76.4	90.2	97.8	98.7	91.0	98.2	77.8	86.7	66.3	82.2	97.8	98.7	91.0	98.2
34	93.3	96.0	83.4	94.7	97.8	98.7	91.0	98.2	87.8	92.7	76.4	90.2	96.7	98.0	88.8	97.3
35	95.6	97.3	86.8	96.4	100.0	100.0	100.0	100.0	90.0	94.0	79.0	92.0	97.8	98.7	91.0	98.2
36	75.6	85.3	64.2	80.4	100.0	100.0	100.0	100.0	83.3	90.0	71.6	86.7	100.0	100.0	100.0	100.0
37	53.3	72.0	46.3	62.7	98.9	99.3	93.9	99.1	57.8	74.7	49.7	66.2	98.9	99.3	93.9	99.1
38	94.4	96.7	85.0	95.6	100.0	100.0	100.0	100.0	97.8	98.7	91.0	98.2	98.9	99.3	93.9	99.1
39	95.6	97.3	86.8	96.4	100.0	100.0	100.0	100.0	96.7	98.0	88.8	97.3	95.6	97.3	86.8	96.4
40	100.0	100.0	100.0	100.0	100.0	100.0	100.0	100.0	83.3	90.0	71.6	86.7	100.0	100.0	100.0	100.0
41	97.8	98.7	91.0	98.2	100.0	100.0	100.0	100.0	85.6	91.3	73.9	88.4	93.3	96.0	83.4	94.7
42	96.7	98.0	88.8	97.3	100.0	100.0	100.0	100.0	93.3	96.0	83.4	94.7	94.4	96.7	85.0	95.6

Table 4: Continued from the previous page.

Material ID	Conv3D No Material				Conv3D All Materials				Spectrogram No Material				Spectrogram All Materials			
	ACC	AUC	F1	Perf.	ACC	AUC	F1	Perf.	ACC	AUC	F1	Perf.	ACC	AUC	F1	Perf.
43	41.1	64.7	37.5	52.9	95.6	97.3	86.8	96.4	45.6	67.3	40.6	56.4	96.7	98.0	88.8	97.3
44	47.8	68.7	42.2	58.2	100.0	100.0	100.0	100.0	54.4	72.7	47.1	63.6	96.7	98.0	88.8	97.3
45	34.4	60.7	32.8	47.6	97.8	98.7	91.0	98.2	36.7	62.0	34.3	49.3	95.6	97.3	86.8	96.4
46	42.2	65.3	38.2	53.8	98.9	99.3	93.9	99.1	52.2	71.3	45.5	61.8	97.8	98.7	91.0	98.2
47	43.3	66.0	39.0	54.7	98.9	99.3	93.9	99.1	48.9	69.3	43.0	59.1	95.6	97.3	86.8	96.4
48	35.6	61.3	33.6	48.4	96.7	98.0	88.8	97.3	43.3	66.0	39.0	54.7	92.2	95.3	81.8	93.8
49	37.8	62.7	35.1	50.2	100.0	100.0	100.0	100.0	44.4	66.7	39.8	55.6	100.0	100.0	100.0	100.0
50	38.9	63.3	35.9	51.1	96.7	98.0	88.8	97.3	38.9	63.3	35.9	51.1	87.8	92.7	76.4	90.2
51	34.4	60.7	32.8	47.6	94.4	96.7	85.0	95.6	36.7	62.0	34.3	49.3	85.6	91.3	73.9	88.4
52	40.0	64.0	36.7	52.0	96.7	98.0	88.8	97.3	40.0	64.0	36.7	52.0	95.6	97.3	86.8	96.4
53	41.1	64.7	37.5	52.9	100.0	100.0	100.0	100.0	44.4	66.7	39.8	55.6	95.6	97.3	86.8	96.4
54	37.8	62.7	35.1	50.2	97.8	98.7	91.0	98.2	44.4	66.7	39.8	55.6	97.8	98.7	91.0	98.2
55	36.7	62.0	34.3	49.3	100.0	100.0	100.0	100.0	46.7	68.0	41.4	57.3	97.8	98.7	91.0	98.2
56	33.3	60.0	32.0	46.7	96.7	98.0	88.8	97.3	41.1	64.7	37.5	52.9	92.2	95.3	81.8	93.8
57	42.2	65.3	38.2	53.8	98.9	99.3	93.9	99.1	50.0	70.0	43.8	60.0	83.3	90.0	71.6	86.7
58	36.7	62.0	34.3	49.3	100.0	100.0	100.0	100.0	41.1	64.7	37.5	52.9	78.9	87.3	67.3	83.1
59	45.6	67.3	40.6	56.4	100.0	100.0	100.0	100.0	54.4	72.7	47.1	63.6	98.9	99.3	93.9	99.1
60	43.3	66.0	39.0	54.7	95.6	97.3	86.8	96.4	46.7	68.0	41.4	57.3	94.4	96.7	85.0	95.6
61	33.3	60.0	32.0	46.7	92.2	95.3	81.8	93.8	40.0	64.0	36.7	52.0	93.3	96.0	83.4	94.7
62	33.3	60.0	32.0	46.7	98.9	99.3	93.9	99.1	51.1	70.7	44.7	60.9	95.6	97.3	86.8	96.4
63	97.8	98.7	91.0	98.2	100.0	100.0	100.0	100.0	95.6	97.3	86.8	96.4	94.4	96.7	85.0	95.6
64	83.3	90.0	71.6	86.7	100.0	100.0	100.0	100.0	78.9	87.3	67.3	83.1	94.4	96.7	85.0	95.6
66	90.0	94.0	79.0	92.0	100.0	100.0	100.0	100.0	94.4	96.7	85.0	95.6	94.4	96.7	85.0	95.6
67	98.9	99.3	93.9	99.1	100.0	100.0	100.0	100.0	86.7	92.0	75.1	89.3	98.9	99.3	93.9	99.1
68	95.6	97.3	86.8	96.4	100.0	100.0	100.0	100.0	91.1	94.7	80.4	92.9	98.9	99.3	93.9	99.1
69	94.4	96.7	85.0	95.6	100.0	100.0	100.0	100.0	95.6	97.3	86.8	96.4	96.7	98.0	88.8	97.3
70	90.0	94.0	79.0	92.0	97.8	98.7	91.0	98.2	90.0	94.0	79.0	92.0	94.4	96.7	85.0	95.6
72	27.8	56.7	28.2	42.2	78.9	87.3	67.3	83.1	22.2	53.3	24.5	37.8	52.2	71.3	45.5	61.8
73	27.8	56.7	28.2	42.2	73.3	84.0	62.3	78.7	16.7	50.0	20.8	33.3	43.3	66.0	39.0	54.7
75	90.0	94.0	79.0	92.0	95.6	97.3	86.8	96.4	93.3	96.0	83.4	94.7	98.9	99.3	93.9	99.1
76	57.8	74.7	49.7	66.2	98.9	99.3	93.9	99.1	61.1	76.7	52.2	68.9	96.7	98.0	88.8	97.3
77	45.6	67.3	40.6	56.4	96.7	98.0	88.8	97.3	64.4	78.7	54.9	71.6	85.6	91.3	73.9	88.4
78	28.9	57.3	29.0	43.1	77.8	86.7	66.3	82.2	21.1	52.7	23.8	36.9	63.3	78.0	54.0	70.7
79	38.9	63.3	35.9	51.1	94.4	96.7	85.0	95.6	42.2	65.3	38.2	53.8	93.3	96.0	83.4	94.7
80	31.1	58.7	30.5	44.9	83.3	90.0	71.6	86.7	32.2	59.3	31.3	45.8	73.3	84.0	62.3	78.7
82	28.9	57.3	29.0	43.1	68.9	81.3	58.5	75.1	17.8	50.7	21.6	34.2	45.6	67.3	40.6	56.4
	**66.8**	**80.1**	**60.3**	**73.4**	**96.8**	**98.1**	**92.8**	**97.5**	**65.8**	**79.5**	**58.3**	**72.7**	**91.6**	**95.0**	**83.6**	**93.3**

Table 5: Gesture classification performance of the Conv3D models trained on synthesized data (data augmentation) and evaluated on materials 1-82, reported Accuracy (ACC), Area Under the ROC Curve (AUC), Micro-F1 score (F1), and the combined performance metric $Perf = (ACC + AUC)/2$. The table continues on the next page.

Material ID	Conv3D RandAugment				Conv3D CCycle GAN				Conv3D Gaussian-noise				Conv3D RandAug + G-Noise			
	ACC	AUC	F1	Perf.	ACC	AUC	F1	Perf.	ACC	AUC	F1	Perf.	ACC	AUC	F1	Perf.
1	40.0	64.0	36.7	52.0	30.0	58.0	29.8	44.0	42.2	65.3	38.2	53.8	63.3	78.0	54.0	70.7
2	93.3	96.0	83.4	94.7	84.4	90.7	72.8	87.6	86.7	92.0	75.1	89.3	75.6	85.3	64.2	80.4
3	93.3	96.0	83.4	94.7	96.7	98.0	88.8	97.3	93.3	96.0	83.4	94.7	83.3	90.0	71.6	86.7
4	95.6	97.3	86.8	96.4	92.2	95.3	81.8	93.8	94.4	96.7	85.0	95.6	84.4	90.7	72.8	87.6
6	94.4	96.7	85.0	95.6	94.4	96.7	85.0	95.6	87.8	92.7	76.4	90.2	74.4	84.7	63.3	79.6
7	97.8	98.7	91.0	98.2	95.6	97.3	86.8	96.4	83.3	90.0	71.6	86.7	78.9	87.3	67.3	83.1
8	85.6	91.3	73.9	88.4	82.2	89.3	70.5	85.8	78.9	87.3	67.3	83.1	72.2	83.3	61.3	77.8
9	98.9	99.3	93.9	99.1	92.2	95.3	81.8	93.8	86.7	92.0	75.1	89.3	81.1	88.7	69.4	84.9
10	26.7	56.0	27.5	41.3	38.9	63.3	35.9	51.1	40.0	64.0	36.7	52.0	68.9	81.3	58.5	75.1
12	95.6	97.3	86.8	96.4	84.4	90.7	72.8	87.6	86.7	92.0	75.1	89.3	76.7	86.0	65.2	81.3
13	100.0	100.0	100.0	100.0	95.6	97.3	86.8	96.4	95.6	97.3	86.8	96.4	84.4	90.7	72.8	87.6
14	90.0	94.0	79.0	92.0	94.4	96.7	85.0	95.6	88.9	93.3	77.7	91.1	80.0	88.0	68.3	84.0
15	94.4	96.7	85.0	95.6	88.9	93.3	77.7	91.1	94.4	96.7	85.0	95.6	75.6	85.3	64.2	80.4
16	94.4	96.7	85.0	95.6	91.1	94.7	80.4	92.9	90.0	94.0	79.0	92.0	78.9	87.3	67.3	83.1
17	94.4	96.7	85.0	95.6	87.8	92.7	76.4	90.2	86.7	92.0	75.1	89.3	73.3	84.0	62.3	78.7
18	24.4	54.7	26.0	39.6	28.9	57.3	29.0	43.1	38.9	63.3	35.9	51.1	68.9	81.3	58.5	75.1
19	84.4	90.7	72.8	87.6	68.9	81.3	58.5	75.1	81.1	88.7	69.4	84.9	76.7	86.0	65.2	81.3
20	82.2	89.3	70.5	85.8	66.7	80.0	56.7	73.3	81.1	88.7	69.4	84.9	74.4	84.7	63.3	79.6
21	51.1	70.7	44.7	60.9	50.0	70.0	43.8	60.0	52.2	71.3	45.5	61.8	57.8	74.7	49.7	66.2
24	98.9	99.3	93.9	99.1	100.0	100.0	100.0	100.0	94.4	96.7	85.0	95.6	83.3	90.0	71.6	86.7
26	65.6	79.3	55.8	72.4	53.3	72.0	46.3	62.7	63.3	78.0	54.0	70.7	67.8	80.7	57.6	74.2
27	87.8	92.7	76.4	90.2	78.9	87.3	67.3	83.1	87.8	92.7	76.4	90.2	75.6	85.3	64.2	80.4
28	91.1	94.7	80.4	92.9	86.7	92.0	75.1	89.3	87.8	92.7	76.4	90.2	78.9	87.3	67.3	83.1
29	88.9	93.3	77.7	91.1	88.9	93.3	77.7	91.1	85.6	91.3	73.9	88.4	66.7	80.0	56.7	73.3
30	98.9	99.3	93.9	99.1	92.2	95.3	81.8	93.8	94.4	96.7	85.0	95.6	80.0	88.0	68.3	84.0
31	91.1	94.7	80.4	92.9	90.0	94.0	79.0	92.0	85.6	91.3	73.9	88.4	78.9	87.3	67.3	83.1
32	85.6	91.3	73.9	88.4	88.9	93.3	77.7	91.1	81.1	88.7	69.4	84.9	71.1	82.7	60.4	76.9
33	86.7	92.0	75.1	89.3	85.6	91.3	73.9	88.4	85.6	91.3	73.9	88.4	68.9	81.3	58.5	75.1
34	96.7	98.0	88.8	97.3	93.3	96.0	83.4	94.7	90.0	94.0	79.0	92.0	76.7	86.0	65.2	81.3
35	91.1	94.7	80.4	92.9	92.2	95.3	81.8	93.8	85.6	91.3	73.9	88.4	72.2	83.3	61.3	77.8
36	90.0	94.0	79.0	92.0	83.3	90.0	71.6	86.7	85.6	91.3	73.9	88.4	63.3	78.0	54.0	70.7
37	65.6	79.3	55.8	72.4	65.6	79.3	55.8	72.4	70.0	82.0	59.4	76.0	70.0	82.0	59.4	76.0
38	100.0	100.0	100.0	100.0	100.0	100.0	100.0	100.0	95.6	97.3	86.8	96.4	84.4	90.7	72.8	87.6
39	98.9	99.3	93.9	99.1	96.7	98.0	88.8	97.3	92.2	95.3	81.8	93.8	83.3	90.0	71.6	86.7
40	94.4	96.7	85.0	95.6	91.1	94.7	80.4	92.9	86.7	92.0	75.1	89.3	78.9	87.3	67.3	83.1
41	96.7	98.0	88.8	97.3	90.0	94.0	79.0	92.0	82.2	89.3	70.5	85.8	76.7	86.0	65.2	81.3
42	95.6	97.3	86.8	96.4	87.8	92.7	76.4	90.2	82.2	89.3	70.5	85.8	72.2	83.3	61.3	77.8

Table 5: Continued from the previous page.

Material ID	Conv3D RandAugment				Conv3D CCycle GAN				Conv3D Gaussian-noise				Conv3D RandAug + G-Noise			
	ACC	AUC	F1	Perf.	ACC	AUC	F1	Perf.	ACC	AUC	F1	Perf.	ACC	AUC	F1	Perf.
43	40.0	64.0	36.7	52.0	57.8	74.7	49.7	66.2	52.2	71.3	45.5	61.8	72.2	83.3	61.3	77.8
44	44.4	66.7	39.8	55.6	61.1	76.7	52.2	68.9	64.4	78.7	54.9	71.6	75.6	85.3	64.2	80.4
45	24.4	54.7	26.0	39.6	43.3	66.0	39.0	54.7	36.7	62.0	34.3	49.3	68.9	81.3	58.5	75.1
46	38.9	63.3	35.9	51.1	63.3	78.0	54.0	70.7	60.0	76.0	51.4	68.0	72.2	83.3	61.3	77.8
47	38.9	63.3	35.9	51.1	60.0	76.0	51.4	68.0	61.1	76.7	52.2	68.9	74.4	84.7	63.3	79.6
48	25.6	55.3	26.7	40.4	52.2	71.3	45.5	61.8	47.8	68.7	42.2	58.2	74.4	84.7	63.3	79.6
49	26.7	56.0	27.5	41.3	60.0	76.0	51.4	68.0	53.3	72.0	46.3	62.7	72.2	83.3	61.3	77.8
50	27.8	56.7	28.2	42.2	38.9	63.3	35.9	51.1	38.9	63.3	35.9	51.1	67.8	80.7	57.6	74.2
51	23.3	54.0	25.3	38.7	50.0	70.0	43.8	60.0	38.9	63.3	35.9	51.1	66.7	80.0	56.7	73.3
52	30.0	58.0	29.8	44.0	48.9	69.3	43.0	59.1	45.6	67.3	40.6	56.4	77.8	86.7	66.3	82.2
53	30.0	58.0	29.8	44.0	44.4	66.7	39.8	55.6	53.3	72.0	46.3	62.7	67.8	80.7	57.6	74.2
54	32.2	59.3	31.3	45.8	60.0	76.0	51.4	68.0	57.8	74.7	49.7	66.2	68.9	81.3	58.5	75.1
55	27.8	56.7	28.2	42.2	48.9	69.3	43.0	59.1	43.3	66.0	39.0	54.7	64.4	78.7	54.9	71.6
56	23.3	54.0	25.3	38.7	34.4	60.7	32.8	47.6	38.9	63.3	35.9	51.1	70.0	82.0	59.4	76.0
57	37.8	62.7	35.1	50.2	47.8	68.7	42.2	58.2	43.3	66.0	39.0	54.7	72.2	83.3	61.3	77.8
58	25.6	55.3	26.7	40.4	44.4	66.7	39.8	55.6	42.2	65.3	38.2	53.8	62.2	77.3	53.1	69.8
59	43.3	66.0	39.0	54.7	57.8	74.7	49.7	66.2	53.3	72.0	46.3	62.7	74.4	84.7	63.3	79.6
60	37.8	62.7	35.1	50.2	61.1	76.7	52.2	68.9	64.4	78.7	54.9	71.6	70.0	82.0	59.4	76.0
61	22.2	53.3	24.5	37.8	21.1	52.7	23.8	36.9	32.2	59.3	31.3	45.8	56.7	74.0	48.8	65.3
62	24.4	54.7	26.0	39.6	47.8	68.7	42.2	58.2	43.3	66.0	39.0	54.7	67.8	80.7	57.6	74.2
63	97.8	98.7	91.0	98.2	91.1	94.7	80.4	92.9	88.9	93.3	77.7	91.1	76.7	86.0	65.2	81.3
64	83.3	90.0	71.6	86.7	87.8	92.7	76.4	90.2	87.8	92.7	76.4	90.2	72.2	83.3	61.3	77.8
66	98.9	99.3	93.9	99.1	94.4	96.7	85.0	95.6	88.9	93.3	77.7	91.1	80.0	88.0	68.3	84.0
67	94.4	96.7	85.0	95.6	94.4	96.7	85.0	95.6	85.6	91.3	73.9	88.4	71.1	82.7	60.4	76.9
68	97.8	98.7	91.0	98.2	94.4	96.7	85.0	95.6	85.6	91.3	73.9	88.4	68.9	81.3	58.5	75.1
69	90.0	94.0	79.0	92.0	91.1	94.7	80.4	92.9	82.2	89.3	70.5	85.8	70.0	82.0	59.4	76.0
70	86.7	92.0	75.1	89.3	94.4	96.7	85.0	95.6	75.6	85.3	64.2	80.4	64.4	78.7	54.9	71.6
72	24.4	54.7	26.0	39.6	18.9	51.3	22.3	35.1	28.9	57.3	29.0	43.1	40.0	64.0	36.7	52.0
73	23.3	54.0	25.3	38.7	16.7	50.0	20.8	33.3	31.1	58.7	30.5	44.9	34.4	60.7	32.8	47.6
75	96.7	98.0	88.8	97.3	90.0	94.0	79.0	92.0	84.4	90.7	72.8	87.6	76.7	86.0	65.2	81.3
76	78.9	87.3	67.3	83.1	83.3	90.0	71.6	86.7	78.9	87.3	67.3	83.1	74.4	84.7	63.3	79.6
77	62.2	77.3	53.1	69.8	72.2	83.3	61.3	77.8	67.8	80.7	57.6	74.2	67.8	80.7	57.6	74.2
78	22.2	53.3	24.5	37.8	16.7	50.0	20.8	33.3	33.3	60.0	32.0	46.7	41.1	64.7	37.5	52.9
79	22.2	53.3	24.5	37.8	42.2	65.3	38.2	53.8	40.0	64.0	36.7	52.0	66.7	80.0	56.7	73.3
80	22.2	53.3	24.5	37.8	16.7	50.0	20.8	33.3	33.3	60.0	32.0	46.7	51.1	70.7	44.7	60.9
82	22.2	53.3	24.5	37.8	16.7	50.0	20.8	33.3	32.2	59.3	31.3	45.8	34.4	60.7	32.8	47.6
	**65.9**	**79.5**	**60.6**	**72.7**	**69.2**	**81.5**	**61.8**	**75.4**	**68.8**	**81.3**	**60.4**	**75.0**	**70.7**	**82.4**	**60.4**	**76.6**

Table 6: Gesture classification performance of the Spectrogram models trained on synthesized data (data augmentation) and evaluated on materials 1-82, reported Accuracy (ACC), Area Under the ROC Curve (AUC), Micro-F1 score (F1), and the combined performance metric $Perf = (ACC + AUC)/2$. The table continues on the next page.

Material ID	Spectro RandAugment				Spectro CCycle GAN				Spectro Gaussian-noise				Spectro RandAug + G-Noise			
	ACC	AUC	F1	Perf.	ACC	AUC	F1	Perf.	ACC	AUC	F1	Perf.	ACC	AUC	F1	Perf.
1	38.9	63.3	35.9	51.1	45.6	67.3	40.6	56.4	41.1	64.7	37.5	52.9	43.3	66.0	39.0	54.7
2	91.1	94.7	80.4	92.9	90.0	94.0	79.0	92.0	87.8	92.7	76.4	90.2	78.9	87.3	67.3	83.1
3	96.7	98.0	88.8	97.3	90.0	94.0	79.0	92.0	95.6	97.3	86.8	96.4	84.4	90.7	72.8	87.6
4	95.6	97.3	86.8	96.4	91.1	94.7	80.4	92.9	88.9	93.3	77.7	91.1	86.7	92.0	75.1	89.3
6	86.7	92.0	75.1	89.3	92.2	95.3	81.8	93.8	87.8	92.7	76.4	90.2	82.2	89.3	70.5	85.8
7	96.7	98.0	88.8	97.3	90.0	94.0	79.0	92.0	96.7	98.0	88.8	97.3	80.0	88.0	68.3	84.0
8	85.6	91.3	73.9	88.4	88.9	93.3	77.7	91.1	84.4	90.7	72.8	87.6	75.6	85.3	64.2	80.4
9	97.8	98.7	91.0	98.2	96.7	98.0	88.8	97.3	95.6	97.3	86.8	96.4	84.4	90.7	72.8	87.6
10	38.9	63.3	35.9	51.1	70.0	82.0	59.4	76.0	54.4	72.7	47.1	63.6	45.6	67.3	40.6	56.4
12	94.4	96.7	85.0	95.6	97.8	98.7	91.0	98.2	88.9	93.3	77.7	91.1	84.4	90.7	72.8	87.6
13	98.9	99.3	93.9	99.1	98.9	99.3	93.9	99.1	94.4	96.7	85.0	95.6	90.0	94.0	79.0	92.0
14	94.4	96.7	85.0	95.6	82.2	89.3	70.5	85.8	88.9	93.3	77.7	91.1	73.3	84.0	62.3	78.7
15	95.6	97.3	86.8	96.4	84.4	90.7	72.8	87.6	85.6	91.3	73.9	88.4	76.7	86.0	65.2	81.3
16	95.6	97.3	86.8	96.4	80.0	88.0	68.3	84.0	87.8	92.7	76.4	90.2	78.9	87.3	67.3	83.1
17	91.1	94.7	80.4	92.9	87.8	92.7	76.4	90.2	85.6	91.3	73.9	88.4	78.9	87.3	67.3	83.1
18	33.3	60.0	32.0	46.7	55.6	73.3	48.0	64.4	45.6	67.3	40.6	56.4	43.3	66.0	39.0	54.7
19	92.2	95.3	81.8	93.8	88.9	93.3	77.7	91.1	77.8	86.7	66.3	82.2	81.1	88.7	69.4	84.9
20	91.1	94.7	80.4	92.9	84.4	90.7	72.8	87.6	83.3	90.0	71.6	86.7	78.9	87.3	67.3	83.1
21	45.6	67.3	40.6	56.4	54.4	72.7	47.1	63.6	50.0	70.0	43.8	60.0	51.1	70.7	44.7	60.9
24	100.0	100.0	100.0	100.0	97.8	98.7	91.0	98.2	93.3	96.0	83.4	94.7	76.7	86.0	65.2	81.3
26	62.2	77.3	53.1	69.8	80.0	88.0	68.3	84.0	74.4	84.7	63.3	79.6	66.7	80.0	56.7	73.3
27	86.7	92.0	75.1	89.3	80.0	88.0	68.3	84.0	84.4	90.7	72.8	87.6	73.3	84.0	62.3	78.7
28	96.7	98.0	88.8	97.3	83.3	90.0	71.6	86.7	87.8	92.7	76.4	90.2	77.8	86.7	66.3	82.2
29	91.1	94.7	80.4	92.9	81.1	88.7	69.4	84.9	83.3	90.0	71.6	86.7	77.8	86.7	66.3	82.2
30	97.8	98.7	91.0	98.2	98.9	99.3	93.9	99.1	88.9	93.3	77.7	91.1	86.7	92.0	75.1	89.3
31	95.6	97.3	86.8	96.4	86.7	92.0	75.1	89.3	91.1	94.7	80.4	92.9	88.9	93.3	77.7	91.1
32	93.3	96.0	83.4	94.7	80.0	88.0	68.3	84.0	85.6	91.3	73.9	88.4	77.8	86.7	66.3	82.2
33	88.9	93.3	77.7	91.1	73.3	84.0	62.3	78.7	76.7	86.0	65.2	81.3	80.0	88.0	68.3	84.0
34	96.7	98.0	88.8	97.3	90.0	94.0	79.0	92.0	95.6	97.3	86.8	96.4	84.4	90.7	72.8	87.6
35	93.3	96.0	83.4	94.7	85.6	91.3	73.9	88.4	93.3	96.0	83.4	94.7	84.4	90.7	72.8	87.6
36	95.6	97.3	86.8	96.4	91.1	94.7	80.4	92.9	93.3	96.0	83.4	94.7	84.4	90.7	72.8	87.6
37	71.1	82.7	60.4	76.9	73.3	84.0	62.3	78.7	76.7	86.0	65.2	81.3	73.3	84.0	62.3	78.7
38	97.8	98.7	91.0	98.2	100.0	100.0	100.0	100.0	94.4	96.7	85.0	95.6	86.7	92.0	75.1	89.3
39	97.8	98.7	91.0	98.2	98.9	99.3	93.9	99.1	90.0	94.0	79.0	92.0	85.6	91.3	73.9	88.4
40	98.9	99.3	93.9	99.1	86.7	92.0	75.1	89.3	93.3	96.0	83.4	94.7	82.2	89.3	70.5	85.8
41	94.4	96.7	85.0	95.6	88.9	93.3	77.7	91.1	90.0	94.0	79.0	92.0	81.1	88.7	69.4	84.9
42	95.6	97.3	86.8	96.4	83.3	90.0	71.6	86.7	88.9	93.3	77.7	91.1	75.6	85.3	64.2	80.4

Table 6: Continued from the previous page.

Material ID	Spectro RandAugment				Spectro CCycle GAN				Spectro Gaussian-noise				Spectro RandAug + G-Noise			
	ACC	AUC	F1	Perf.	ACC	AUC	F1	Perf.	ACC	AUC	F1	Perf.	ACC	AUC	F1	Perf.
43	54.4	72.7	47.1	63.6	75.6	85.3	64.2	80.4	62.2	77.3	53.1	69.8	62.2	77.3	53.1	69.8
44	66.7	80.0	56.7	73.3	86.7	92.0	75.1	89.3	73.3	84.0	62.3	78.7	68.9	81.3	58.5	75.1
45	30.0	58.0	29.8	44.0	62.2	77.3	53.1	69.8	45.6	67.3	40.6	56.4	52.2	71.3	45.5	61.8
46	54.4	72.7	47.1	63.6	83.3	90.0	71.6	86.7	72.2	83.3	61.3	77.8	68.9	81.3	58.5	75.1
47	64.4	78.7	54.9	71.6	87.8	92.7	76.4	90.2	76.7	86.0	65.2	81.3	74.4	84.7	63.3	79.6
48	38.9	63.3	35.9	51.1	64.4	78.7	54.9	71.6	58.9	75.3	50.5	67.1	54.4	72.7	47.1	63.6
49	50.0	70.0	43.8	60.0	81.1	88.7	69.4	84.9	64.4	78.7	54.9	71.6	70.0	82.0	59.4	76.0
50	40.0	64.0	36.7	52.0	64.4	78.7	54.9	71.6	57.8	74.7	49.7	66.2	47.8	68.7	42.2	58.2
51	34.4	60.7	32.8	47.6	64.4	78.7	54.9	71.6	52.2	71.3	45.5	61.8	52.2	71.3	45.5	61.8
52	42.2	65.3	38.2	53.8	82.2	89.3	70.5	85.8	60.0	76.0	51.4	68.0	71.1	82.7	60.4	76.9
53	51.1	70.7	44.7	60.9	72.2	83.3	61.3	77.8	66.7	80.0	56.7	73.3	64.4	78.7	54.9	71.6
54	55.6	73.3	48.0	64.4	76.7	86.0	65.2	81.3	63.3	78.0	54.0	70.7	66.7	80.0	56.7	73.3
55	38.9	63.3	35.9	51.1	71.1	82.7	60.4	76.9	57.8	74.7	49.7	66.2	60.0	76.0	51.4	68.0
56	32.2	59.3	31.3	45.8	64.4	78.7	54.9	71.6	57.8	74.7	49.7	66.2	47.8	68.7	42.2	58.2
57	54.4	72.7	47.1	63.6	78.9	87.3	67.3	83.1	63.3	78.0	54.0	70.7	54.4	72.7	47.1	63.6
58	43.3	66.0	39.0	54.7	76.7	86.0	65.2	81.3	62.2	77.3	53.1	69.8	61.1	76.7	52.2	68.9
59	54.4	72.7	47.1	63.6	90.0	94.0	79.0	92.0	72.2	83.3	61.3	77.8	68.9	81.3	58.5	75.1
60	56.7	74.0	48.8	65.3	76.7	86.0	65.2	81.3	72.2	83.3	61.3	77.8	68.9	81.3	58.5	75.1
61	18.9	51.3	22.3	35.1	50.0	70.0	43.8	60.0	37.8	62.7	35.1	50.2	41.1	64.7	37.5	52.9
62	33.3	60.0	32.0	46.7	80.0	88.0	68.3	84.0	56.7	74.0	48.8	65.3	63.3	78.0	54.0	70.7
63	96.7	98.0	88.8	97.3	87.8	92.7	76.4	90.2	92.2	95.3	81.8	93.8	83.3	90.0	71.6	86.7
64	91.1	94.7	80.4	92.9	73.3	84.0	62.3	78.7	81.1	88.7	69.4	84.9	74.4	84.7	63.3	79.6
66	97.8	98.7	91.0	98.2	87.8	92.7	76.4	90.2	94.4	96.7	85.0	95.6	78.9	87.3	67.3	83.1
67	96.7	98.0	88.8	97.3	83.3	90.0	71.6	86.7	91.1	94.7	80.4	92.9	81.1	88.7	69.4	84.9
68	84.4	90.7	72.8	87.6	84.4	90.7	72.8	87.6	90.0	94.0	79.0	92.0	78.9	87.3	67.3	83.1
69	87.8	92.7	76.4	90.2	83.3	90.0	71.6	86.7	85.6	91.3	73.9	88.4	86.7	92.0	75.1	89.3
70	87.8	92.7	76.4	90.2	83.3	90.0	71.6	86.7	88.9	93.3	77.7	91.1	83.3	90.0	71.6	86.7
72	16.7	50.0	20.8	33.3	17.8	50.7	21.6	34.2	16.7	50.0	20.8	33.3	17.8	50.7	21.6	34.2
73	16.7	50.0	20.8	33.3	16.7	50.0	20.8	33.3	16.7	50.0	20.8	33.3	18.9	51.3	22.3	35.1
75	96.7	98.0	88.8	97.3	100.0	100.0	100.0	100.0	94.4	96.7	85.0	95.6	93.3	96.0	83.4	94.7
76	78.9	87.3	67.3	83.1	97.8	98.7	91.0	98.2	83.3	90.0	71.6	86.7	75.6	85.3	64.2	80.4
77	73.3	84.0	62.3	78.7	85.6	91.3	73.9	88.4	75.6	85.3	64.2	80.4	70.0	82.0	59.4	76.0
78	16.7	50.0	20.8	33.3	16.7	50.0	20.8	33.3	16.7	50.0	20.8	33.3	22.2	53.3	24.5	37.8
79	38.9	63.3	35.9	51.1	54.4	72.7	47.1	63.6	56.7	74.0	48.8	65.3	54.4	72.7	47.1	63.6
80	16.7	50.0	20.8	33.3	21.1	52.7	23.8	36.9	16.7	50.0	20.8	33.3	25.6	55.3	26.7	40.4
82	16.7	50.0	20.8	33.3	16.7	50.0	20.8	33.3	16.7	50.0	20.8	33.3	18.9	51.3	22.3	35.1
	**70.4**	**82.3**	**63.9**	**76.3**	**77.1**	**86.2**	**68.0**	**81.7**	**73.3**	**84.0**	**64.4**	**78.6**	**68.8**	**81.3**	**59.5**	**75.1**
